# Anti-PD-1 treatment response is associated with the influx of circulating myeloid and T-cell subsets into the metastatic melanoma tumor microenvironment

**DOI:** 10.1038/s41416-025-03137-8

**Published:** 2025-09-02

**Authors:** S. Van Dam, D. Krijgsman, E. Küçükköse, M. E. L. Verdonschot, M. Amini, W. A. M. Blokx, M. J. M. Van Eijs, R. J. Verheijden, O. Kranenburg, K. P. M. Suijkerbuijk, J. H. W. Leusen, Y. Vercoulen

**Affiliations:** 1 Center for Molecular Medicine, University Medical Center Utrecht, Utrecht University, Utrecht, The Netherlands; 2 Center for Translational Immunology, University Medical Center Utrecht, Utrecht University, Utrecht, The Netherlands; 3 Laboratory Translational Oncology, Division of Imaging and Cancer, University Medical Center Utrecht, Utrecht University, Utrecht, The Netherlands; 4 UCyTOF, Center for Molecular Medicine, University Medical Center Utrecht, Utrecht University, Utrecht, The Netherlands; 5 Department of Pathology, University Medical Center Utrecht, Utrecht University, Utrecht, The Netherlands; 6 Department of Medical Oncology, University Medical Center Utrecht, Utrecht University, Utrecht, The Netherlands

**Keywords:** Molecular medicine, Immunotherapy, Imaging the immune system, Predictive markers, Melanoma

## Abstract

**Background:**

Immune checkpoint inhibition (ICI) significantly improves the survival of metastatic melanoma patients; however, a substantial proportion of patients does not respond to these breakthrough therapies.

**Methods:**

To improve our understanding of this response variability, we developed high-plex panels for protein imaging of a discovery cohort and validation with RNAseq analyses to examine myeloid and T-cell subsets in pre-anti-PD-1-treatment samples of 14 metastatic melanoma patients (7 responders and 7 non-responders).

**Results:**

We demonstrate that a higher abundance of circulating monocyte-derived macrophages (MDMs) and cytotoxic T-cell subsets in the tumor microenvironment (TME) at baseline distinguishes metastatic melanoma patients with a favorable response to anti-PD1 treatment from non-responders, who featured co-localization of suppressive macrophages (M2) and T-cells. Additionally, MDMs expressed high levels of immune checkpoints, and MDM infiltration into the TME was linked to both ICI response and survival.

**Conclusion:**

These findings highlight the potential of MDM infiltration as a predictive biomarker for ICI response in metastatic melanoma.

## Background

Immune checkpoint inhibitors (ICIs) represent a breakthrough treatment of advanced (i.e. irresectable metastatic) melanoma, as the use of anti-PD-1 anti-CTLA-4 combination therapy increases median overall survival (OS) from 6-9 months to nearly six years [1–4]. However, more than half of the patients treated with ICIs targeting PD-1/PD-L1 and/or CTLA-4 do not respond, and patients can development drug resistance [5–8]. This failure of immunotherapeutic strategies may be attributed to a suppressive immune landscape in the tumors and T-cell dysfunction, also described as exhaustion, within the TME [9]. In general, TME includes a wide range of T-cell subsets, such as T_H_1 cells, regulatory T-cells (T_regs_), (resident) memory T-cells (T_RM_ and T_MEM_), progenitor exhausted T-cells (TPEX) and terminally exhausted T-cells (TEX), with each subset impacting the anti-tumor response differently. For example, in patients that respond to anti-PD-1 and anti-CTLA-4 combination therapy, the presence of TPEX is correlated with prolonged progression-free survival (PFS) and overall survival (OS) [10]. Profound immunosuppression in the TME is also mediated by fibroblasts, T_regs_, tumor associated macrophages and suppressive myeloid cells, including macrophages and myeloid-derived suppressor cells (MDSCs) [8, 11]. Suppressive myeloid cells are present in the TME of most patients and are potent inhibitors of T-cell-mediated anti-tumor responses [12]. Therefore, targeting the myeloid compartment has emerged as a promising approach in combination with existing therapeutic strategies.

MDSCs comprise an immature myeloid immune cell subset that expands under pathological conditions, including inflammation and cancer [13, 14]. Two major subsets of MDSCs have been identified: polymorphonuclear MDSCs (PMN-MDSCs, CD11b^+^CD33^+^CD14^-^CD15^+^LOX-1^+^) and monocytic MDSCs (M-MDSCs, CD11b^+^CD33^+^CD14^+^HLA-DR^-/low^CD15^-^) [15–17]. MDSCs suppress T-cell activation and cytotoxicity and drive T-cell and macrophage polarization towards T_regs_ and macrophages with a suppressive phenotype (M2), respectively. In addition, circulating monocytes in metastatic melanoma patients may contribute to tumor progression in part by mediating tumor-induced immunosuppression [18]. When infiltrating into the tissue, monocytes differentiate into macrophages with distinct phenotypes and functions. M1 macrophages are involved in pro-inflammatory responses, while M2 macrophages play a role in anti-inflammatory processes and tumor progression in melanoma patients [19]. Finally, monocyte-derived macrophages (MDM) that represent a transition state as monocytes differentiate into macrophages have also been described. Immune suppressive mechanisms used by myeloid cells include the production of reactive oxygen species (ROS) [20] and induction of L-arginine depletion via nitric oxide (NO) and arginase 1 production [21]. Furthermore, MDSCs suppress T-cell activation via expression of immunosuppressive checkpoint molecules, including PD-L1 and VISTA [22, 23], thereby abrogating the effect of current ICI treatment strategies that are focused on T-cell activation in the TME.

In this study, we explored the myeloid and T-cell immune landscape of metastatic melanoma pre-treatment samples from ICI responders and non-responders using imaging mass cytometry (IMC). Hereto, we developed synergistic myeloid and T-cell panels that allow for high-plex IMC and deep phenotyping of cell subsets infiltrating the tumor. By assessing the suppressive myeloid TME and their interactions with T-cells, we aim to better understand the composition of these tumors in the context of ICI response to optimize treatment strategies for melanoma patients in the future.

## Methods

### Study population and clinical data collection

This study retrospectively included 15 advanced-stage non-uveal melanoma patients (stage IV) who received first-line anti-PD-1 (pembrolizumab or nivolumab) monotherapy for ≥ 12 weeks at the University Medical Center Utrecht (UMCU), the Netherlands. Response was evaluated based on RECIST 1.1 criteria as complete response (CR), partial response (PR), stable disease (SD), or progressive disease (PD) [24]. The best overall response within the treatment episode was used for analyses. No pseudo-progression was observed within the included patients. Clinical data including patient demographics (sex, age), treatment regimen, clinical response, PFS, and OS were collected from patient records (Supplementary Table 1 and Supplementary Table 2). PFS and OS were defined as time from the first date of anti-PD-1 treatment until progression or death. The Dutch Medical Research Ethical Committee did not consider the study subject to the Dutch Medical Research with Human Subjects Law. The study received approval from the Biobank Committee of the UMCU (Tc-bio #18-123). Permission to use tissue from this biobank in the present study was obtained through material release protocol TC-bio 18-397. All participants provided written informed consent in line with the Declaration of Helsinki.

### Sample selection

Formalin-fixed paraffin-embedded (FFPE) tumor tissue samples were collected between 2015 and 2020. From all patients, baseline samples were obtained via surgical tumor resection or biopsy.

An expert pathologist, blinded for the treatment response, annotated three 500 × 500 µm regions of interest (ROIs) per sample based on the presence of viable tumor cells and immune infiltrate in the center of the tumor. Included lymph node biopsies contained large tumor areas. The pathologist only selected tumor regions with immune infiltrate within these large tumor areas to prevent analysis of pre-existent immune cells that are present in the lymph node. Tertiary lymphoid structures and immune infiltrate around vessels were not selected during annotation.

### Antibody panels, antibody conjugation and validation

The antibodies employed in the myeloid and T-cell panels are detailed in Supplementary Table 3 and 4, respectively. For IMC, carrier-free antibodies were conjugated with metal isotopes following the manufacturer’s protocol using the MaxPar X8 labeling kit from Fluidigm. These isotope-conjugated antibodies were subsequently stored in antibody stabilizer phosphate-buffered saline (PBS, Boca Scientific) at a concentration of 500 μg/ml at 4 °C until needed. Antibodies were tested prior to conjugation using immunofluorescence according manufacturer's instructions. Tissues for IF testing were selected using the Human Protein Atlas. After conjugation, antibodies were tested in different dilutions on the selected tissue of the human protein atlas and tumor tissue. An overview of all the antibody panels on tumor tissue is detailed in Supplementary Figs. 1 and 2.

### IMC sample preparation

Four-micrometer sections of FFPE tumor tissues were mounted on glass slides (Klinipath) and subjected to an hour of baking at 60 °C. Sequential sections were employed for staining with the myeloid and T-cell panels, respectively, following established IMC staining procedures [25, 26]. For the myeloid panel, tissue sections were incubated overnight at 4 °C in a humidified chamber with anti-SIRPα in Tris Buffered Saline with Tween (TBST) containing 0.5% Bovine Serum Albumin (BSA). Subsequently, after three 5-minute washes in TBST, the sections were exposed to a secondary rabbit-161Dy antibody in TBST containing 0.5% BSA for 1 hour at room temperature (RT). Following another set of three 5-minute TBST washes, tissue sections were incubated overnight at 4 °C in a humidified chamber with a mixture of metal-labeled antibodies in TBST containing 0.5% BSA, as outlined in Supplementary Table 3. For the T-cell panel, tissue sections were incubated overnight at 4 °C in a humidified chamber with anti-CTLA-4 in TBST containing 0.5% BSA. After subsequent washing with TBST, samples were treated with a secondary rabbit-154Sm antibody in TBST containing 0.5% BSA for 1 hour at RT. After a final set of three 5-minute TBST washes, tissue sections were exposed to a mixture of metal-labeled antibodies in TBST containing 0.5% BSA, as specified in Supplementary Table 4, for overnight incubation at 4 °C.

The next day, the samples underwent three 5-minute TBST washes and were incubated with DAPI (Sigma-Aldrich, 1:10.000 dilution in TBST/0.5% BSA). Following two ddH2O rinses, slides were allowed to air dry and subsequently imaged via fluorescence microscopy. Post-imaging, slides were initially immersed in TBST for 10 min. Subsequently, they were subjected to a 5 min incubation in 1% Toluidine blue (Merck) and a 5 min ddH2O rinse. Slides were then exposed to the DNA intercalator Ir193 (Fluidigm, 1:1000 dilution in TBST) for 1 h at RT. Finally, slides were washed in TBST for 5 min and allowed to air dry.

### IMC single-cell data generation

The MATISSE pipeline was used to process fluorescence and IMC images to single-cell data as previously described [25, 26]. Cells were segmented in Cellprofiler with the following settings in the myeloid panel and T cell panels: Threshold strategy: Global; Thresholding method: Otsu; Threshold smoothing scale: 0.5 (myeloid)/0.0 (T cell); Threshold correction factor: 1.0; Lower and upper bounds on thresholding: 0–1.0 (myeloid)/0–0.2 (T cell); Regularization factor: 0.05). A tissue microarray (TMA) was included on every glass slide, containing control tissue of different origins, which were also stained and ablated and used to inspect for experimental variation between slides. 99th percentile normalization was used for data normalization. Single-cell data was log1p transformed for clustering and expression analyses in R (version 4.1.2). Tissues from patient 8 were excluded from analyses due to insufficient tissue quality, characterized by extensive necrosis that resulted in poor nuclear staining and, consequently, inadequate cell segmentation.

### Downstream analyses of the myeloid panel

Single-cell clusters were generated with the Rphenograph package in R, based on the mean expression per cell of the markers: MPO, CD33, CD16, CD15, CD68, FOXP3, CD20, CD4, CD45, CD3, CD8a, CD14, HLA-DRDPDQ, CD11b, and SOX10. The number of nearest neighbors was set at 100 for clustering. Clusters were then assigned to myeloid, lymphoid, or nonimmune classes and reclustered using nearest neighbors set at 300. Reclustering of myeloid cells was based on: CD14, CD68, CD15, CD11b, MPO, CD16, CD47, VISTA, IL-10, pERK, pSTAT3, NFκb, SIRPα, PD-L1, PD-1, LOX-1, CD163, HLA-DRDPDQ, CD123, Arginase 1, and iNOS. Reclustering of lymphoid cells was performed using CD3, CD4, CD8a, FOXP3, CD20, PD-1, VISTA, Granzyme B, HLA-DRDPDQ, CD16, and IFNγ. Finally, nonimmune cells were reclustered using SOX10, CD10, Ki67, HLA-DRDPDQ, PD-L1, and CD47. Uniform Manifold Approximation and Projection (UMAP) was performed using the UMAP package. CytoMAP was used for neighborhood analysis using a raster scanned approach with a radius of 50 µm [27].

### Downstream analyses of the T-cell panel

IMC data were scaled and log-transformed. To enable detection of T cell subsets based on biologically relevant markers in the literature—including both surface markers and transcription factors—we employed an alternative strategy, in which cell lineages were annotated as previously described [28]. In short, modal intensities were used to calculate scaling factors for each channel and each patient separately. Data was linearly scaled, aligning channel-specific single-cell expression distributions among patients. To ensure robust and reproducible data normalization, we visually checked scaling results using histogram representations and images of single-cell data across patients.

Afterwards, specific levels of positivity were established for markers selected to determine lineage as described. For markers with bimodal expression, a threshold distinguishing marker-positive and -negative populations was applied. In the case of unimodal markers where the true positive signal was contained in the right-heavy tail, we employed the full width at half maximum (FWHM) method to derive a normal distribution from the left half of the histogram, conservatively setting a positivity threshold at +1.5 standard deviations (SD). Using the Boolean rules stated in Supplementary Table 5, candidate cell type annotations were created based on markers identifying different subsets. The expression levels of key markers (indicated in bold in the table) were ranked across all cells from all patients combined. For each individual cell, the rankings of the key markers were compared between the candidate cell types. The key marker with the highest rank ultimately determined the assigned cell type label for each individual cell. Given the limitations of imaging resolution, individual single-cell signals inevitably contained some degree of signal from neighboring cells, which complicated unsupervised annotation based purely on membrane markers. To address this, we applied a supervised approach in which multiple candidate cell types were initially assigned per cell, followed by a comparative ranking of key marker expression levels. The highest-ranked key marker ultimately determined the final cell type classification. Cells that did not meet the criteria for any of the cell types but were CD45^+^ were labeled as “other immune cells,” and cells that lacked CD45^+^ were labeled as “non-classified.”

### Transcriptome analysis

The RNAseq processed count data (PRJEB23709) of Gide et al. and single-cell RNAseq data from Pozniak et al. were analyzed using the R2: Genomics Analysis and Visualization Platform (http://r2.amc.nl) [29, 30]. The ‘monocyte-derived-macrophage’ [31] and ‘Macrophage’ [32] signatures were used. We used the following genes for the ‘M1’ signature: *IL6, CXCL8, CD80, PIM1, RTP4, SLC11* *A1, NOS2, CD40*, and *CD86*. The genes for the ‘M2’ signature are: *CD163, MRC1, ARG1, IL4I1, IL13*. No gene signatures were available for the chemokine and cytokine analysis. Hence, single gene analysis was done for: *CSF1, CSF1R, CCL2, CCL5, CCR7, IL34, CX3CL1, CXCL9, CXCL10, CXCL13, CXCL17 (bulk)* and within specific CD4^+^, CD8^+^ T cell, monocyte and macrophage immune cell subsets as defined by UMAP clustering *(single cell RNAseq)*.

### Statistical analyses

Mann-Whitney U tests were used to compare cell types between responders and non-responders. Marker expression was compared by a Mann-Whitney U test with Benjamini-Hochberg correction. The Pearson correlation test was used for calculating the correlation between cell abundancies and for neighborhood analysis. Neighborhood analyses were normalized on a per-ROI basis and normalized for cell abundance. PFS and OS were analyzed using Kaplan-Meier survival curves and compared using the log-rank test. Patients were censored for PFS and OS analyses according to Gide et al. [30]. P < 0.05 was considered statistically significant. Figures were generated using FIJI (v2.9.0/1.53t), Graphpad Prism (v9), and Adobe Illustrator [29] (v. 25.0). Schematic illustration were created using Biorender.com.

## Results

### Patient characteristics and study design

**Fig. 1:**
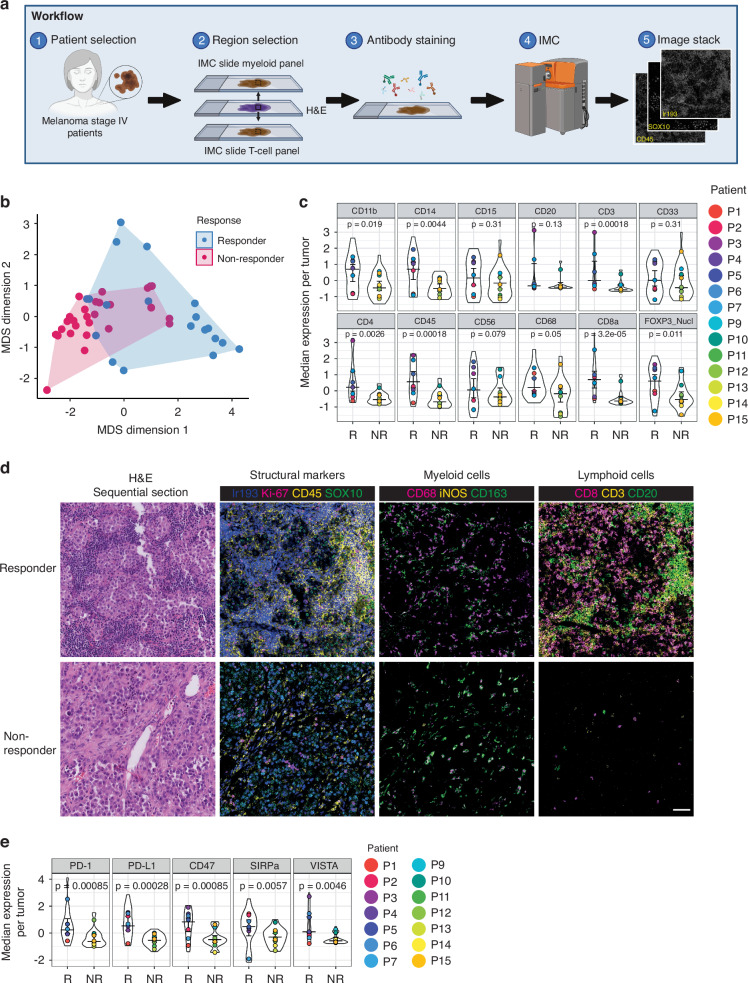
High plex imaging mass cytometry to characterize the immune landscape of metastatic melanoma. **a** Illustration of the data acquisition workflow used for IMC: (1) tumor tissue derived from metastatic melanoma tissue was obtained prior to the start of anti-PD-1 treatment. (2) Three 500 × 500 μm regions of interest (ROI) were selected based on H&E-stained sections by a pathologist and used on sequential tissue sections for IMC stainings. (3) Tissue sections were stained with a myeloid or T-cell panel consisting of metal isotope-labeled antibodies. (4) ROI was ablated with a high-energy laser using IMC. (5) Each antibody resulted in a single image per sample with the metal isotope composition per pixel, together constructing a multi-channel image stack. **b** Multidimensional scaling (MDS) using the median intensity of each marker per image region as input variables. Each data point is an ROI, color-coded by tumor state. **c** The median intensity of each lineage marker was calculated per ROI. The violin plots show the median intensity of each lineage marker per patient (across ROIs) split for therapy response. Mann-Whitney U-test with Benjamini-Hochberg correction was used to calculate the statistical difference between non-responders and responders. Bars indicate the median with a 95% confidence interval. **d** Representative H&E and IMC images. The scale bar is 50 μm. **e** The median intensity of each immune checkpoint was calculated per ROI. The violin plots show the median intensity of each lineage marker per patient (across ROIs) split for therapy response. Patient ID is indicated in colors. Mann-Whitney U-test with Benjamini-Hochberg correction was used to calculate the statistical difference between non-responders and responders. Bars indicate the median with a 95% confidence interval.

To comprehensively study the myeloid and T-cell immune landscape in metastatic melanoma, we included 15 patients receiving anti-PD-1 monotherapy that exhibited a wide range of clinical response. One patient (patient 8) was excluded from analyses due to insufficient tissue quality. Thus, we analyzed metastatic tissue samples from 14 anti-PD1 treated patients using two customized IMC panels on consecutive sections focused on myeloid and T-cells, respectively (Fig. 1a). Patient characteristics are summarized in Supplementary Table 1 and 2. Among the 14 included patients with advanced melanoma, 11 patients (79% of the cohort) received pembrolizumab, and 3 patients (21%) received nivolumab. Out of the total patients, 7 patients (50%) had a radiological response and 7 patients (50%) did not respond. A BRAF driving mutation was present in 57% of the responding patients and in 43% of the non-responding patients. Responding patients had a median PFS and OS of 70 months, and non-responding patients had a median PFS of 3 months and a median OS of 21 months. In total, 47 regions of interest (ROIs) with immune infiltration were annotated by the pathologist. The myeloid IMC panel encompassed 47 ROIs (20 ROIs of 8 responding and 27 ROIs of 7 non-responding), with a total of 84,022 cells analyzed (Supplementary Table 6). The T-cell IMC panel encompassed 44 ROIs (19 ROIs of 8 responding and 25 ROIs of 7 non-responding), with a total of 119,295 cells analyzed (Supplementary Table 6). Three ROIs from three different patients were excluded since the DAPI images were not present for image registration. Taken together, our cohort was diverse from a perspective of the type of the first-line anti-PD-1 therapy received (nivolumab or pembrolizumab) and patient response (responders and non-responders) and allowed us to collect and analyze both myeloid and T-cell IMC, collectively covering more than 200,000 cells.

### Response to immunotherapy is correlated with an immune hot phenotype

First, we set out to compare the overall expression signatures based on the markers in the myeloid panel, and multidimensional scaling (MDS) of median expression levels per ROI revealed distinct clustering of therapy response groups (Fig. 1b). Responders exhibited significantly higher expression of immune marker CD45 (*p* < 0.001) and T-cell markers CD3 (*p* < 0.001), CD4 (*p* = 0.0026), CD8a (*p* < 0.001), and FOXP3 (*p* = 0.011) compared to non-responders (Fig. 1c, Supplementary Fig. 3). Higher expression of myeloid markers CD11b (*p* = 0.019), CD14 (*p* = 0.0044), and CD68 (*p* = 0.050) was also observed in responders, albeit to a lesser extent. Representative IMC images are shown in Fig. 1d. Additionally, responders displayed elevated expression of immune checkpoints PD-1 (*p* < 0.001), PD-L1 (*p* < 0.001), CD47 (*p* < 0.001), SIRPα (*p* = 0.0057), and VISTA (*p* = 0.0046) compared to non-responders (Fig. 1e). Overall, our data confirm previous data showing that immunotherapy response is associated with the “hot tumor” phenotype (immune cell type-rich tumors), which has a high immune cell density in the tumor [33, 34]. Although non-responders showed limited T-cell infiltration, myeloid infiltration was still evident.

### Increased myeloid compartment in non-responders

**Fig. 2:**
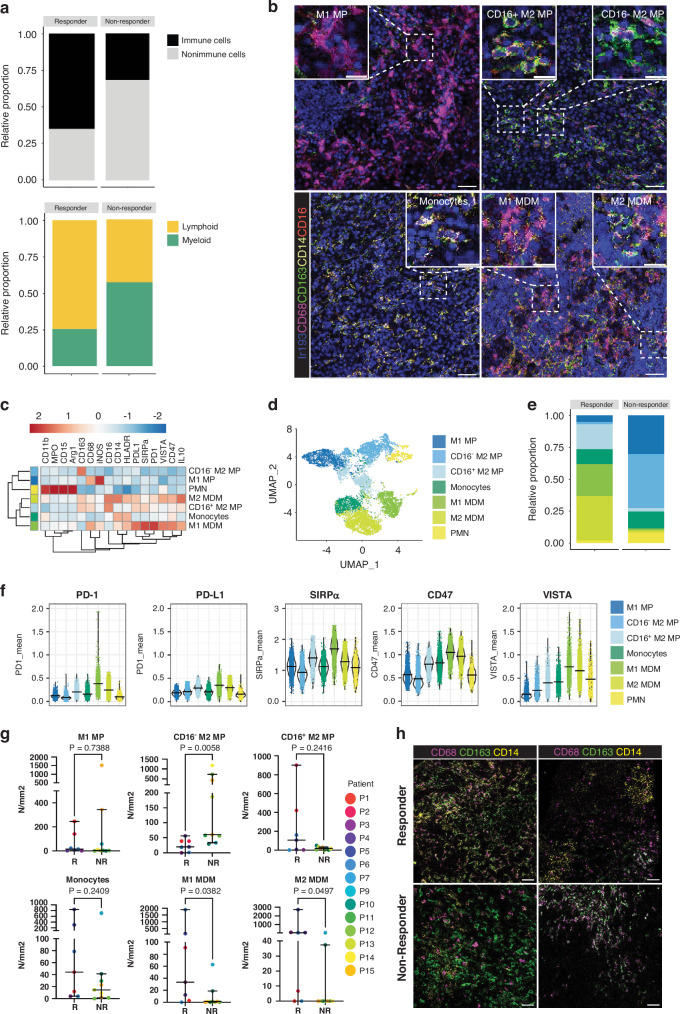
Shift in the myeloid compartment in non-responders compared to responders. **a** Stacked bar plot representing the relative proportions of annotated immune cells versus non-immune cells and myeloid versus lymphoid cell types, stratified by therapy response. **b** Representative IMC images indicating the different identified myeloid cell types. The scale bar in overview images is 50 μm. The scale bar is 20 μm in inserts. **c** Hierarchical clustering heatmap of the median marker intensity per patient indicated for the 7 annotated myeloid cell types. **d** Uniform Manifold Approximation and Projection (UMAP) of single cells of all ROIs combined using lineage marker intensities, color-coded by annotated myeloid cell lineages. **e** Stacked bar plot representing the proportions of annotated myeloid cell types stratified by therapy response. **f** Violin plots indicating the median expression of immune checkpoints in the identified myeloid cell clusters. Every dot is a single cell. Cell type is indicated in colors. Bars indicate the median with a 95% confidence interval. **g** Dot plots of the median abundance of annotated myeloid cell clusters, stratified by therapy response. Each dot is a patient. The bars indicate the median with a 95% confidence interval. Nonparametric T-tests were used to calculate the statistical difference between non-responders and responders. **h** Representative IMC images illustrating a high abundance of M2 MP (CD68^+^CD163^+^) in non-responders and a high abundance of MDM (CD68^+^CD14^+^) in responders. The scale bar is 50 μm.

Next, we assessed the distribution of immune cells in metastatic melanoma and conducted unsupervised clustering. The mean relative abundance of immune cells in responders (65.0%) was significantly higher compared to non-responders (31.7%, *p* = 0.0229) (Fig. 2a), although patient-specific variations were observed (Supplementary Fig. 4). Overall, the mean relative abundance of myeloid cells was higher in non-responders (57.4%) compared to responders (25.8%, *p* = 0.0229) (Fig. 2A), consistent with findings from a recent study by Schlenker et al. [35]. However, the absolute number of total myeloid cells did not differ between responders and non-responders (Supplementary Figs. 4 and 5). To further investigate this disparity in therapeutic response, we conducted a comprehensive analysis of myeloid and lymphoid subsets within this patient cohort, aiming to identify potential underlying mechanisms.

We distinguished three distinct macrophage subsets: ‘M1 MP’ (CD68^+^iNOS^+^CD163^-^), ‘CD16^+^ M2 MP’ (CD68^+^iNOS^-^CD163^+^CD16^+^), and ‘CD16^−^ M2 MP’ (CD68^+^iNOS^-^CD163^+^CD16^-^) (Fig. 2b–e). Furthermore, we identified a monocyte population (CD14^+^) and a group containing polymorphonuclear cells (PMN). Given that the PMN subset exhibited varying expression of the suppressive markers arginase 1 and myeloperoxidase (MPO), it is likely to include both active PMN, and suppressive PMN-MDSC (Supplementary Fig. 6). Two subpopulations co-expressing CD14 and CD68, indicative of MDMs, were defined based on CD163 expression as ‘M1 MDM’ (CD14^+^CD68^+^CD163^−^) and ‘M2 MDM’ (CD14^+^CD68^+^CD163^+^), in alignment with the M1/M2 definitions used for macrophages. Examples of identified macrophages, MDMs, and monocytes are shown in Fig. 2b Importantly, monocytes and both MDM populations displayed elevated phosphorylated ERK (pERK) expression (Supplementary Fig. 6), a pivotal factor in their differentiation into macrophages, which is in agreement with previous studies showing that pERK can be induced through colony-stimulating factor 1 (CSF-1 or M-CSF) mediated MAPK signaling [36].

Notably, immune checkpoints PD-1, PD-L1, SIRPα, CD47, and VISTA were expressed in CD16^+^ M2 macrophages, monocytes, and both MDM populations, albeit at varying levels (Fig. 2f). In contrast, M1 macrophages, CD16^-^ M2 macrophages, and PMN generally displayed lower immune checkpoint expression. Upon closer examination, it became evident that immune checkpoint expression consistently manifested at higher levels in CD16^+^ M2 macrophages, monocytes, and both MDM populations among responders when compared to non-responders (Supplementary Fig. 7). In summary, the melanoma immune infiltrate in non-responders contained relatively more myeloid cells compared to that of responders. While the same subsets were present in both groups, their expression of immune checkpoints was lower in non-responders.

### The myeloid compartment contains more M2 macrophages but reduced MDM in non-responders

Remarkably, we observed a shift within the myeloid compartment, with a higher relative (Fig. 2e) and absolute (Fig. 2g) abundance of ‘CD16^-^ M2 MP’ in non-responders (*p* = 0.0058). However, no significant differences were found in ‘M1 MP’ (*p* = 0.7388), ‘CD16^+^ M2 MP’ (*p* = 0.2416), and ‘Monocytes’ (*p* = 0.2409) between the two groups. Conversely, responders exhibited significantly higher numbers of ‘M1 MDM’ (*p* = 0.0382) and ‘M2 MDM’ (*p* = 0.0497). By contrast, no discernible differences were observed in the abundance of PMN between responders and non-responders (data not shown). It is important to note, however, that while these general trends were observed, there is considerable variability between individual patients, as highlighted in Supplementary Fig. 4C. Figure 2h displays representative IMC images showcasing the high presence of M2 macrophages in non-responders and the high presence of MDM populations in responders. Together, while the TME in non-responders contained more suppressive M2 macrophages, tumors from responders displayed an increased influx of MDM before ICI-therapy. This suggests a potential role for alterations in myeloid infiltration and a more pronounced MDM phenotype within the TME, which may either influence or serve as a predictor of the ICI response.

### Cytotoxic CD4^+^ and CD8^+^ T-cell subsets are enriched in responders

**Fig. 3:**
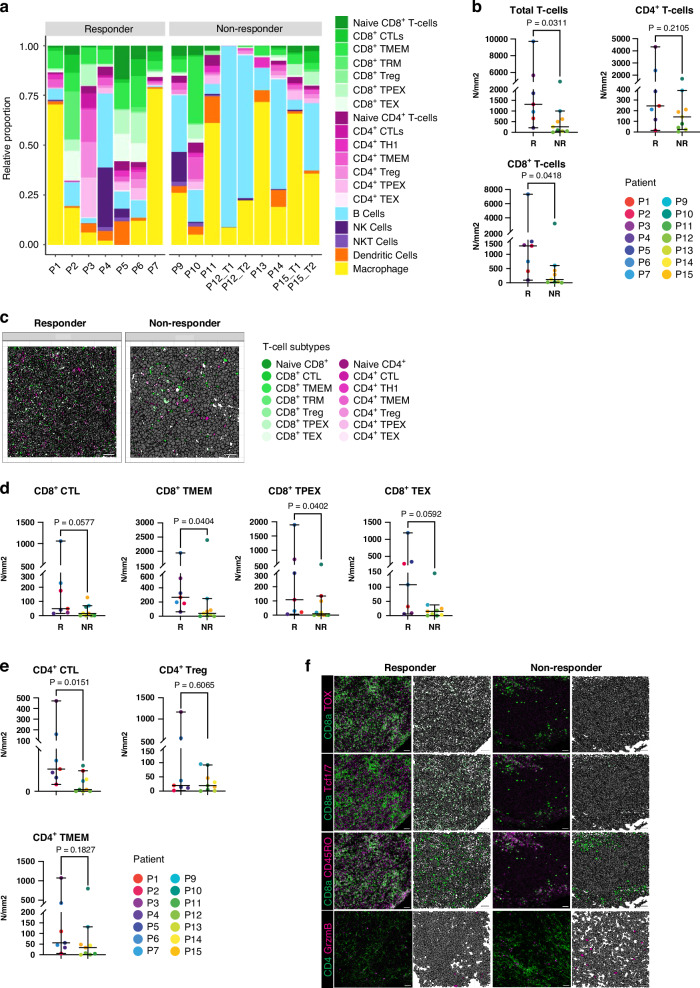
Differences in T-cell abundance in metastatic melanoma. **a** Stacked bar plot representing the proportions of annotated CD4^+^ T-cell subsets (pink) and CD8^+^ T-cell subsets (green) and other identified immune subsets stratified by therapy response. **b** Dot plots of the median abundance of annotated CD4^+^ T-cells and CD8^+^ T-cells stratified for responder (R) and non-responder (NR). Each dot is a patient. The bars indicate the median with a 95% confidence interval. Mann-Whitney U-test was used to calculate the statistical difference between non-responders and responders. **c** Representative confetti images illustrating CD4^+^ T-cell subsets (pink) and various CD8^+^ T-cell subsets (green) in responders compared to non-responders. Scale bar is 40 μm. **d** Dot plots of the median abundancy of annotated CD8^+^ T-cells subsets including naive CD8^+^ T-cells, cytotoxic CD8^+^ T-cells (CD8^+^ CTL), regulatory CD8^+^ T-cells (CD8^+^ T_reg_), CD8^+^ tissue resident T-cells (CD8^+^ T_RM_), CD8^+^ memory T-cells (CD8^+^ T_MEM_), CD8^+^ progenitor exhausted T-cells (CD8^+^ TPEX), and CD8^+^ terminally exhausted T-cells (CD8^+^ TEX), stratified for responder (R) and non-responder (NR). Each dot is a patient. The bars indicate the median with a 95% confidence interval. Mann-Whitney U-test was used to calculate the statistical difference between non-responders and responders. **e** Dot plots of the median abundancy of annotated CD4^+^ T-cells subsets, including naive CD4^+^ T-cells, cytotoxic CD4^+^ T-cells (CD4^+^ CTL), regulatory CD4^+^ T-cells (CD4^+^ T_reg_), CD4^+^ memory T-cells (CD4^+^ T_MEM_), CD4^+^ progenitor exhausted T-cells (CD4^+^ TPEX), and CD4^+^ terminally exhausted T-cells (CD4^+^ TEX), stratified by responder (R) and non-responder (NR). Each dot is a patient. The bars indicate the median with a 95% confidence interval. Mann-Whitney U-test was used to calculate the statistical difference between non-responders and responders. **f** Representative IMC images (left) of CD8^+^ TEX (indicated by TOX in magenta and CD8a in green), CD8^+^ TPEX (indicated by TCF1/7 in magenta and CD8a in green), CD8^+^ T_MEM_ (indicated by CD45RO in magenta and CD8a in green), and CD4^+^ CTL (indicated by Granzyme B in magenta and CD4 in green). Confetti images (right) illustrating CD4+ T-cell subsets (pink) and various CD8+ T-cell subsets (green). The scale bar is 40 μm.

To assess presence of different functional T-cell subsets, we applied a marker-based annotation method that allows separation of biologically relevant subsets [28]. We identified various functional infiltrating CD4^+^ and CD8^+^ T-cell subsets in the TME, displaying inter-patient variation (Fig. 3a, Supplementary Fig. 8A). Despite this variation, we found that tumors of responders contained significantly more T-cell infiltrate compared to non-responders, as anticipated (Fig. 3b, c). This difference could be attributed to a significant increase in CD8^+^ T-cell infiltrate (*p* = 0.0418), while overall CD4^+^ T-cell numbers were similar (Fig. 3b). Next, we explored the different CD4^+^ and CD8^+^ T-cell subsets in tumor infiltrates to identify potential differences associated with treatment response. CD8^+^ T-cell subset analysis revealed that responders had significantly more CD8^+^ T-cell memory cells (T_MEM_) (*p* = 0.0404) and progenitor exhausted CD8^+^ T-cells (CD8^+^ TPEX) (*p* = 0.0402) (Fig. 3d, f, Supplementary Fig. 8B). Additionally, a higher abundancy of CD8^+^ cytotoxic T-cells (CD8^+^ CTL) (p = 0.0577) and terminally exhausted T-cells (CD8^+^ TEX) (*p* = 0.0592) were present in responders. While total CD4^+^ T-cell abundance was not related to therapy response, CD4^+^ cytotoxic T-cells (CD4^+^ CTL) were elevated in responders (*p* = 0.0151) (Fig. 3e, f, Supplementary Fig. 8C). Overall, despite substantial inter-patient variability, tumors from ICI responders showed higher pre-treatment abundance of activated CD8^+^ and CD4^+^ cytotoxic T-cell subsets.

### Distinct expression patterns of exhaustion markers in CD4^+^ and CD8^+^ TPEX and TEX

**Fig. 4:**
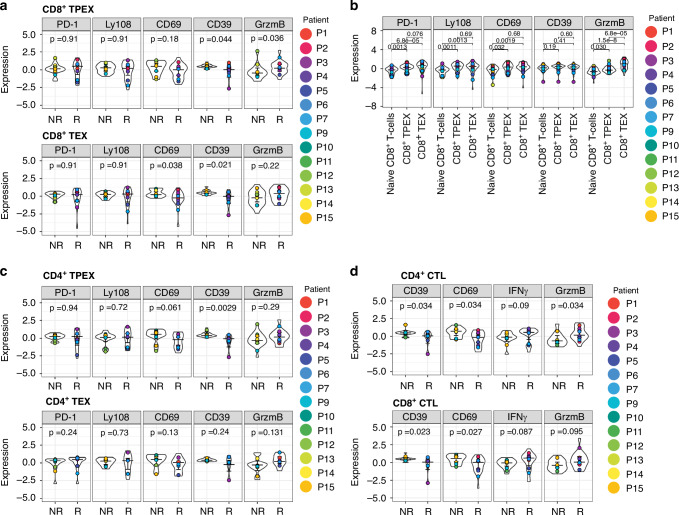
Expression of various markers in TPEX, TEX, CTL CD4^+^ and CD8^+^ T-cell subsets. **a** Violin plot with the median intensity of PD-1, Ly108, CD69, CD39, and Granzyme B (GrzmB) in CD8^+^ TPEX and CD8^+^ TEX cells for responders (R) and non-responders (NR). Patient ID is indicated in colors. Mann-Whitney U-test with Benjamini-Hochberg correction was used to calculate the statistical difference between non-responders and responders. Bars indicate the median with a 95% confidence interval. **b** Violin plot with the median intensity of PD-1, Ly108, CD39, CD69, and Granzyme B (GrzmB) in CD8^+^ naive T-cells, CD8^+^ TPEX and CD8^+^ TEX cells. Patient ID is indicated in colors. Mann-Whitney U-test with Benjamini-Hochberg correction was used to calculate the statistical difference between non-responders and responders. Bars indicate the median with a 95% confidence interval. **c** Violin plot with the median intensity of PD-1, Ly108, CD39, CD69 and Granzyme B (GrzmB) in CD4^+^ TPEX and CD4^+^ TEX cells for responders (R) and non-responders (NR). Patient ID is indicated in colors. Mann-Whitney U-test with Benjamini-Hochberg correction was used to calculate the statistical difference between non-responders and responders. Bars indicate the median with a 95% confidence interval. **d** Violin plot with the median intensity of CD39, CD69, IFNγ and Granzyme B (GrzmB) in CD4^+^ CTL and CD8^+^ CTL-cells for responders (R) and non-responders (NR). Patient ID is indicated in colors. Mann-Whitney U-test was with Benjamini-Hochberg correction was used to calculate the statistical difference between non-responders and responders. Bars indicate the median with a 95% confidence interval.

PD-1 expression has been established as a hallmark for T-cell exhaustion. While showing inter-patient variation, PD-1 was significantly higher expressed by both CD8^+^ TPEX cells and CD8^+^ TEX cells compared to naive CD8^+^ T-cells (CD8^+^ TPEX *p* = 0.0013 and CD8^+^ TEX *p* < 0.001), but did not differ in expression in TPEX and TEX cell subsets between responders and non-responders (Fig. 4a, b) [37]. Ly108 and CD69, relevant for tissue residency, have been linked to T-cell exhaustion development in experimental models of chronic infection and cancer [38]: TPEX cells (Ly108^+^CD69^+^) can transition via Ly108^-^CD69^-^ to Ly108^-^CD69^+^ TEX. Although this transition was not fully recapitulated in our dataset due to variable expression levels, we found that CD8^+^ TPEX cells showed increased Ly108 expression (*p* = 0.0011) (Fig. 4b, Supplementary Fig. 9), while CD8^+^ TEX cells displayed significantly elevated CD69 expression compared to naive CD8^+^ T-cells (*p* = 0.0019). Overall, while expression of Ly108 in exhausted T-cell subsets was similar, CD69 expression was lower in CD8^+^ TEX (*p* = 0.038) from responders, suggesting potentially reduced T-cell tissue residency in ICI responders (Fig. 4a).

Expression of the immunosuppressive endonuclease CD39 has been associated with tumor-infiltrating T-cell exhaustion [39]. We found that CD8^+^ TEX cells and CD8^+^ TPEX cells expressed variable, but lower levels of CD39 in responders (CD8^+^ TPEX: *p* = 0.044, CD8^+^ TEX: *p* = 0.021) (Fig. 4a), suggesting reduced immune suppression [40]. In the TME, CD8^+^ TEX can still express the cytotoxic molecule Granzyme B [41]. In concordance with published data, the CD8^+^ TPEX (*p* = 0.036), but not CD8^+^ TEX (*p* = 0.22), expressed more Granzyme B in ICI responders (Fig. 4a). Expression of Granzyme B was higher in both CD8^+^ TEX (*p* < 0.001) and CD8^+^ TPEX (*p* < 0.001) expressed compared to naive CD8^+^ T-cells (Fig. 4b), suggesting an altered cytotoxic profile in the TME of ICI responders [41].

### Infiltrating CD4^+^ T-cells show parallels with CD8^+^ T-cell exhaustion profiles

To uncover potential features of CD4^+^ T-cell dysfunction in cancer and parallels with CD8^+^ T-cell exhaustion, we used the same marker profiles to investigate CD4^+^ T-cell subsets, which were identified by the expression of Tcf1/7 and TOX, respectively [10, 42]. Like in CD8^+^ subsets, PD-1 is significantly increased in CD4^+^ TEX (*p* = 0.013) compared to CD4^+^ naive T-cells. (Supplementary Fig. 8D).

Tissue residency markers showed comparable patterns in CD4^+^ T-cells as well: While Ly108 expression did not differ, CD69 expression was variable, but overall lower in CD4^+^ TPEX in responders (*p* = 0.061) (Fig. 4c). CD39 expression was significantly reduced (*p* = 0.0029) in CD4^+^ TPEX but not in CD4^+^ TEX (*p* = 0.24) in responders (Fig. 4c). In summary, CD4^+^ T-cell subsets show overlapping phenotypical and functional characteristics with CD8^+^ TEX and TPEX, including reduced CD39 and elevated Granzyme B in ICI responders.

### CD4^+^ CTL are more abundant and display elevated markers for cytotoxic activity in responders

We additionally investigated the features of CD4^+^ CTLs (Supplementary Fig. 10A, B), which have been identified based on gene signatures in solid tumors and associated with positive clinical outcomes [43, 44]. Interestingly, we observed significantly elevated levels of CD4^+^ CTLs (*p* = 0.0151) and a trend towards increased CD8^+^ CTLs (*p* = 0.0577) in responders (Fig. 3d, e). Of note, this difference could be attributed to part of the responder samples displaying high numbers, while others were similar to non-responders. Both CD4^+^ and CD8^+^ CTLs in responders expressed significantly less CD39 (CD4^+^ CTLs: *p* = 0.034, CD8^+^ CTLs: *p* = 0.023) and CD69 (CD4^+^ CTLs: *p* = 0.034, CD8^+^ CTLs: *p* = 0.027) (Fig. 4d). Moreover, in responders, CD4^+^ CTLs expressed higher levels of Granzyme B (*p* = 0.034) (Fig. 4d). Taken together, tumors from some of the responders contained more CD4^+^ CTLs that overall produced higher levels of Granzyme B and lower CD39 and CD69 than in non-responders.

### Decreased tumor-specific T_MEM_ cells with cytotoxicity in responders

Since T_MEM_ cells contribute to antitumor immunity [45], we evaluated the CD8^+^ and CD4^+^ T_MEM_ phenotypes. While CD8^+^ T_MEM_ cell infiltration was increased in responders, the numbers of CD4^+^ T_MEM_ did not relate to response (Fig. 3d, e). However, the functional phenotype of CD4^+^ T_MEM_ was variable, yet distinct between responders and non-responders. CD4^+^ T_MEM_ cells, but not CD8^+^ T_MEM_ cells, had somewhat elevated levels of Granzyme B (*p* = 0.056) in responders (Supplementary Fig. 8E). Again, in responders we observed significantly reduced CD39 expression in both CD4^+^ and CD8^+^ T_MEM_ (CD4^+^ T_MEM_: *p* = 0.012, CD8^+^ T_MEM_: *p* = 0.0056) (Supplementary Fig. 8E). Additionally, CD8^+^ T_MEM_ cells expressed higher pS6 levels in responders (*p* = 0.046), showing active signal transduction (Supplementary Fig. 8E).

Overall, infiltrating CD8^+^ T_MEM_ numbers were increased, and T_MEM_ subsets demonstrated a more active phenotype in ICI responders. Both CD4^+^ T_MEM_ and CD8^+^ T_MEM_ displayed reduced expression of CD39 in responders, like other infiltrating T-cell subsets.

### MDM infiltration associates with improved survival

**Fig. 5:**
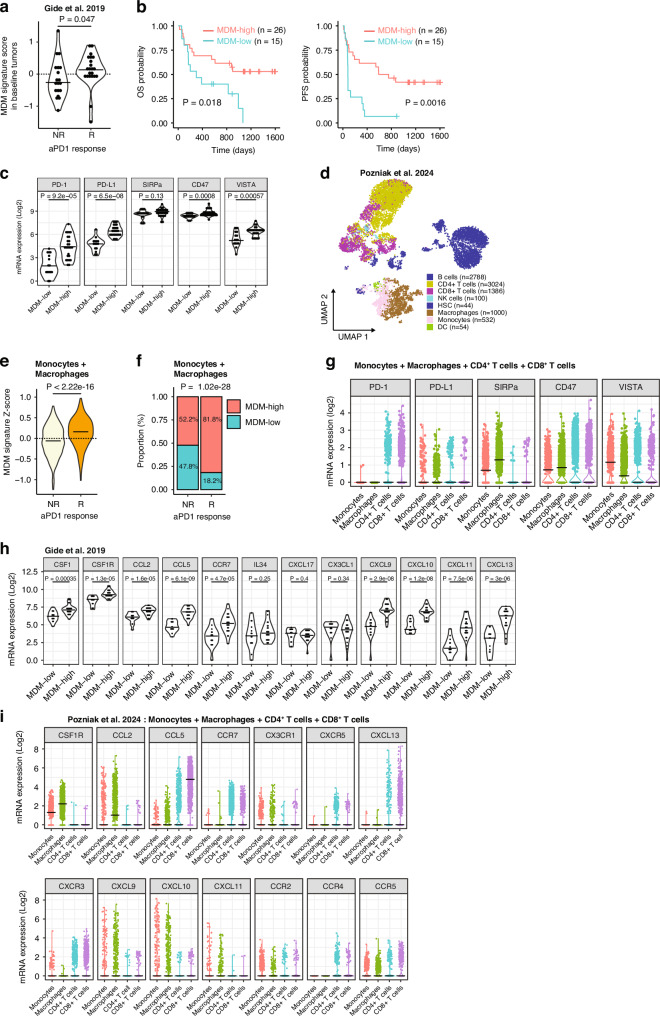
Presence of MDM associates with positive ICI response and prolonged survival. **a** Violin plots illustrating the Z-scores of the ‘monocyte-derived-macrophage’ (MDM) signature [31] in tumors of non-responders (NR) and responders (R) prior to anti-PD-1 treatment [30]. Mann-Whitney U-test was applied. **b** Kaplan–Meier curves demonstrate overall survival (OS, left) and progression-free survival (PFS) (right) in tumor subgroups of anti-PD-1 pre-treatment samples of metastatic melanoma patients characterized by the MDM signature using the k-means algorithm. A two-sided log-rank test was applied. **c** Violin plots illustrate the messenger RNA (mRNA) expression (log2-transformed RPM) of immune checkpoint-related genes (PD-1 (*PDCD1*), PD-L1 (*CD274*), SIRPα (*SIRPA*), CD47 (*CD47*), and VISTA (*VSIR)* in MDM^High^ and MDM^Low^ tumors of metastatic melanoma patients prior to anti-PD-1 treatment. Mann-Whitney U-test was applied. **d** UMAP of single immune cell transcriptome profiles prior to anti-PD-1 treatment from metastatic melanoma patients (5 responders, 11 non-responders) [29]. **e** Violin plot illustrating the Z-scores of the MDM signature [31] in monocytes and macrophages prior to anti-PD-1 treatment. Mann-Whitney U-test was applied. **f** Proportion of MDM^Low^ and MDM^High^ (characterized using the k-means algorithm) in monocytes and macrophages. Chi-squared test was applied. **g** Violin plots illustrate the mRNA expression (log2-transformed RPM) of immune checkpoint-related genes (PD-1 (*PDCD1*), PD-L1 (*CD274*), SIRPα (*SIRPA*), CD47 (*CD47*), and VISTA (*VSIR)* in monocytes, macrophages, CD4^+^ T cells, and CD8^+^ T cells. **h** Violin plots illustrate the mRNA expression (log2-transformed RPM) of cytokines and chemokine genes (*CSF1, CSFR1, CCL2, CCL5, CCR7, IL34, CXCL17, CX3CL1, CXCL9, CXCL10, CXCL11, CXCL13*) in MDM^High^ and MDM^Low^ tumors of metastatic melanoma patients prior to anti-PD-1 treatment [30]. Mann-Whitney U-test was applied. **i**. Violin plots illustrate the mRNA expression (log2-transformed RPM) of cytokines and chemokine genes (*CSF1R, CCL2, CCL5, CCR7, CX3CR1, CXCR5, CXCL13, CXCR3, CXCL9, CXCL10, CXCL11, CCR2, CCR4, CCR5*) in monocytes, macrophages, CD4^+^ T cells, and CD8^+^ T cells [29].

Our analyses revealed differences in myeloid and T-cell abundance and functionality between responders and non-responders. For the first time, we identified elevated MDM infiltration in tumors of ICI responders. Subsequently, we aimed to further explore and validate these observations with therapeutic outcomes using a comprehensive bulk RNAseq dataset comprising 73 pre-treatment samples from patients with advanced melanoma who underwent anti-PD-1, or anti-PD-1 and CTLA-4 combination therapy (total *n* = 73; *n* = 41 anti-PD-1) [30, 46]. Notably, in agreement with our IMC analysis, responders exhibited a significantly elevated expression of the MDM gene signature compared to non-responders (*p* = 0.014) (Fig. 5a, Supplementary Fig. 11A). Next, we generated MDM^Low^ and MDM^High^ subgroups using the k-means algorithm. Of note, the MDM^High^ subgroup included elevated CD16 and CD163 expression, indicating similarity to the CD163^+^ MDM subset (Supplementary Fig. 11B). The OS and PFS were significantly better for MDM^High^ patients compared to MDM^Low^ patients (Fig. 5b, Supplementary Fig. 11C). The presence of macrophages was elevated in responders and was associated with better PFS but not OS. Both M1 and M2 macrophages were equally elevated in responders, but overall, the presence of these macrophages did not differentiate between responders and non-responders (Supplementary Fig. 11D–F). This is in contrast with our IMC analyses which revealed a higher number of M2 macrophages in non-responders.

Interestingly, immune checkpoint transcriptome analysis in 41 anti-PD-1 pre-treatment samples revealed significant upregulation of *VSIR* (VISTA, *p* < 0.001), *CD47* (*p* < 0.001), *PDCD1* (PD-1, *p* < 0.001), and *CD274* (PD-L1, *p* < 0.001) in MDM^High^ tumors compared to MDM^Low^ tumors (Fig. 5c).

Next, we validated our findings in a single-cell RNA sequencing dataset of pre-treatment melanoma samples, allowing separation of different immune cell subsets for analysis (Fig. 5d) [29]. Analyses of myeloid subsets (monocytes and macrophages) confirmed that the MDM signature score was higher in anti-PD-1 responders (Fig. 5e). This was further supported by the large proportion (82%) of myeloid cells with a MDM^High^ signature in responders, compared to non-responders (52%) (Fig. 5f).

Assessment of checkpoint expression demonstrated that PD-1 was almost exclusively expressed by CD4^+^ and CD8^+^ T-cells, PD-L1 was expressed by both T- and myeloid subsets, and SIRPα, CD47 and VISTA were highest expressed in myeloid cells (Fig. 5g).

This suggests that MDM, predominantly found in responsive patients, could support the response to T-cell targeted anti-PD-1 therapy. Additionally, the analyses revealed the presence of myeloid immune checkpoints *VSIR* (VISTA) and *CD47* on MDM, suggesting that these may represent potential additional therapeutic targets.

### Chemokines that attract myeloid and T-cells are elevated in MDM-infiltrated tumors

We observed that ICI responders have an influx of MDM; therefore, we analyzed transcriptomes of cytokines and chemokines that are involved in myeloid trafficking into the tumor. In tumors with an MDM^High^ signature, *CSF1* (p < 0.001), *CSF1R* (*p* < 0.001), *CCL2* (*p* < 0.001), *CCL5* (*p* < 0.001), and *CCR7* (*p* < 0.001) mRNA levels were significantly increased compared to MDM^Low^ tumors.

Additionally, we evaluated the mRNA levels of T-cell recruiting chemokines, such as *CXCL9*, *CXCL10*, *CXCL11* and *CXCL13* which is involved in TLS formation [47]. We observed a significant increase of *CXCL9* (*p* < 0.001), *CXCL10* (p < 0.001), *CXCL11* (*p* < 0.001), and *CXCL13* (*p* < 0.001) mRNA in pre-ICI treatment samples of MDM^High^ tumors, and in responders compared to responders (Fig. 5h, Supplementary Fig. 11G). Furthermore, *CXCL9* (*p* < 0.001), *CXCL10* (*p* < 0.001), *CXCL11* (*p* < 0.001), and *CXCL13* (*p* < 0.001) are significantly elevated in MDM^High^ tumors compared to MDM^Low^ tumors (Fig. 5h, Supplementary Fig. 11G). Single-cell analyses revealed that CSF1R and CCL2, were indeed expressed by macrophages and monocytes, while CD8^+^ T cells produced CCL5 (Fig. 5i). On the other hand, T-cell recruiting chemokines CXCL9, 10, and 11, were highly produced by both macrophages and monocytes, indicating that the myeloid cells infiltrating melanoma can attract T-cells and vice-versa. Together, mRNA levels of chemokines that attract myeloid and T-cells into the TME are elevated in responders, which also have an MDM^High^ signature (Supplementary Fig. 11H, I**)**.

### Neighborhood analysis reveals co-localization between suppressive macrophages and T-cells

**Fig. 6:**
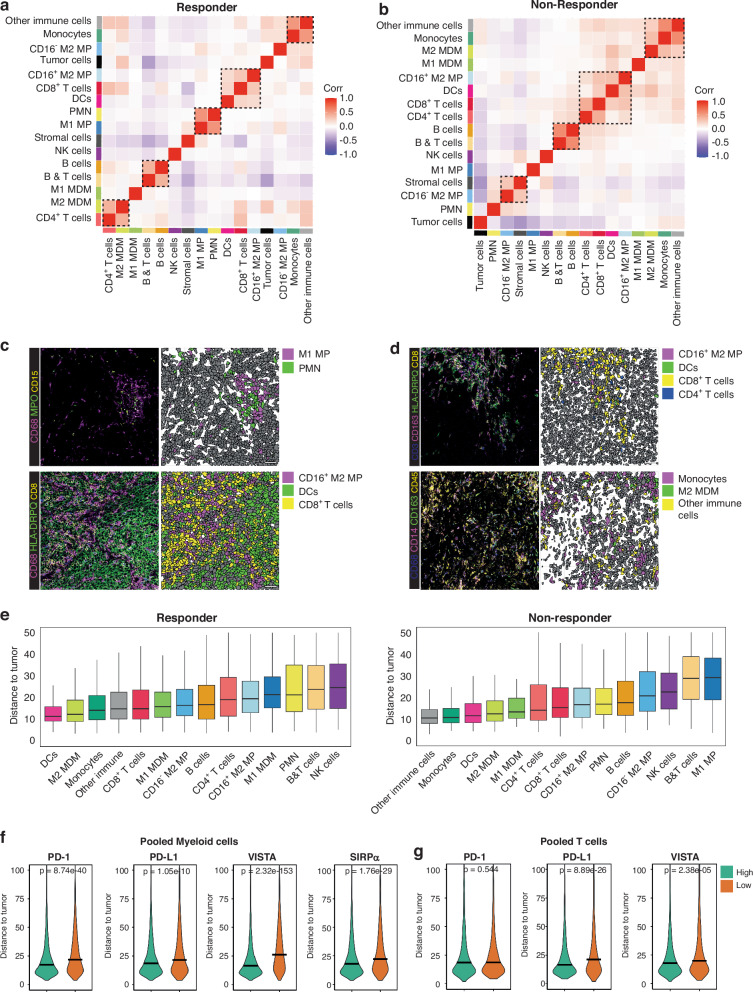
Neighborhood analysis identifies co-localization between T-cells and myeloid cells in metastatic melanoma, and high immune checkpoint expression on myeloid cells close to tumor cells. **a** Correlation heatmap showing the correlation of spatial localization of myeloid cell types within 50 μm raster-based defined neighborhoods in responders. Colors on the borders of the plot indicate the annotated cell lineages. Pearson correlation tests indicate avoidance (blue) or co-localization (red) of cellular localization. The boxes indicate specific correlation patterns which are explained in the text. **b** Correlation heatmap showing the correlation of spatial localization of myeloid cell types within 50 μm raster-based defined neighborhoods in non-responders. Colors on the borders of the plot indicate the annotated cell lineages. Pearson correlation tests indicate avoidance (blue) or co-localization (red) of cellular localization. The boxes indicate specific correlation patterns which are explained in the text. **c** Examples of IMC images and single-cell segmented confetti plots highlighting key interactions in the immune landscape of anti-PD-1-responding metastatic melanomas, as shown in  (**a**). The scale bar is 50 μm. **d** Examples of IMC images and single-cell segmented confetti plots highlighting key interactions in the immune landscape of anti-PD-1-responding metastatic melanomas, as shown in (**b**). The scale bar is 50 μm. **e** Bar graphs illustrating the distance of immune cell subsets to the nearest tumor cell. The median distance to tumor cells in shown, including the interquartile range. **f** Violin plots of the distance of pooled myeloid cells to the nearest tumor cell, split by high (above-median) or low (below-median) checkpoint expression. The median is shown. **g** Violin plots of the distance of pooled T cells to the nearest tumor cell, split by high (above-median) or low (below-median) checkpoint expression. The median is shown.

To investigate how myeloid and T-cell co-localization may relate to therapy response, we conducted a comprehensive spatial neighborhood analysis to explore co-localization between cell subsets (Fig. 6a, b). An overview with the spatial distribution of immune subsets per ROI is included in Supplementary Fig. 12. Initially, we observed co-localization between B cells and B & T-cells in both responder and non-responder samples, confirming their overlapping populations. Additionally, co-localization was evident between monocytes and ‘other immune cells’ in both groups (Fig. 6a, c). The subset with other immune cells, comprising a small number of cells, is likely composed of DCs, as the myeloid panel did not include a specific DC marker. Consequently, DCs were identified CD45^+^HLADRDPPQ^+^ cells; however, this method of identification does not capture the entire DC population. Of particular interest was the co-localization observed between PMN and M1 macrophages in responders (Fig. 6a, c). PMN with high arginase 1 expression (presumably PMN-MDSCs) are recognized for their suppressive capabilities and their potential to induce M2 macrophage polarization, suggesting an active process at play in responders. Intriguingly, this co-localization was notably absent in non-responders, who, in general, exhibited a higher presence of M2 MP (Fig. 6b). This suggests that factors beyond cell-cell interactions, such as environmental cytokines and chemokines within the TME, may contribute to macrophage polarization in non-responders. Furthermore, both responders and non-responders exhibited co-localization of CD16^+^ M2 macrophages with dendritic cells (DCs) and CD8^+^ T-cells in responders (Fig. 6a, c) and CD4^+^ and CD8^+^ T-cells in non-responders (Fig. 6b, d).

Next, we investigated which specific T-cell subtypes co-localize with myeloid cells (Supplementary Fig. 13A, B). In responders, macrophages did not co-localize with most other cell types but co-localized with specific T-cell subsets, such as CD4^+^ TEX, CD8^+^ CTL, CD8^+^ T_RM_, and CD8^+^ T_MEM_ (Supplementary Fig. 13A). Moreover, both specific CD4^+^ subsets (CD4^+^ Treg and CD4^+^ T_MEM_), and CD8^+^ subsets clustered (CD8^+^ TEX, Naive CD8^+^ T-cells, CD8^+^ T_MEM_, and CD8^+^ CTL) co-localized, implicating organized clustering of CD4^+^ subsets and CD8^+^ subsets within the TME. In non-responders, overall localization was more random: T-cell subset co-localization was mixed without clear clusters of subsets, with boxes in Supplementary Fig. 13B indicating highest scores of co-localization, including between CD8^+^ and CD4^+^ PTEX and T_MEM_ cells. Furthermore, in non-responders, DCs, CD8^+^ TEX cells and NKT cells co-localized (Supplementary Fig. 13B). Furthermore, in non-responders, dendritic cells, CD8^+^ TEX cells, and NKT cells co-localized (Supplementary Fig. 13B), whereas in responders only NK cells and NKT cells exhibited co-localization (Supplementary Fig. 13A). In summary, responders exhibit a more organized TME with co-localization of CD16^+^ M2 macrophages, DCs, and CD8^+^ T-cells. Notably, non-responders have a higher abundancy of M2 macrophages, aligning with their immunosuppressive activity.

### High immune checkpoint expression on myeloid cells near tumor cells

We next examined the proximity of specific immune cell subsets to tumor cells and whether any differences existed between responders and non-responders. In both groups, DCs, MDMs, and monocytes were found to be closest to tumor cells (Fig. 6e), even though tumor cells were scarcer in responders. Neighborhood analyses revealed no significant interactions between DCs/MDMs/Monocytes and tumor cells (Fig. 6a, b), suggesting that while these cell types are in close proximity to tumor cells, they also co-localize with other immune cells, without a preferential association with tumor cells.

We were interested in whether the variation in immune checkpoint expression observed within immune cell subsets (Figs. 2f, 4b, Supplementary Fig. 8D) was correlated with the positioning of these immune cells relative to tumor cells. Therefore, we categorized all myeloid and T cells based on low (below-median) and high (above-median) immune checkpoint expression levels and assessed their proximity to tumor cells. Notably, myeloid cells with high expression of PD-1, PD-L1, VISTA, and SIRPα were significantly closer to tumor cells compared to those with low expression of these checkpoints (Fig. 6f). A similar trend was observed for T cells regarding PD-L1 and VISTA expression (Fig. 6g), although no significant difference in proximity to tumor cells was observed for PD-1 expression. These findings suggest that the upregulation of immune checkpoints, such as PD-1, PD-L1, VISTA, and SIRPα, in myeloid cells is associated with their closer proximity to tumor cells.

## Discussion

In this study, we explored the immunological landscape in metastatic melanoma samples before treatment from both patients that responded to anti-PD-1 treatment and those that did not. We employed a comprehensive approach, conducting bulk and single-cell analyses at both mRNA and protein levels. We focused particularly on subsets of myeloid and T-cells infiltrating the tumor tissue, leading to the identification of distinct immune cell composition, checkpoint expression, and inflammatory profiles in responding and non-responding patients. Consistent with existing literature [48], responders exhibited an increased immune infiltrate. Notably, this difference was mainly attributed to a higher proportion of lymphoid cells in responders, while non-responders exhibited a relatively higher abundance of myeloid cells. Importantly, the classification of myeloid cells remains a complex and challenging task, and we acknowledge the inherent complexity and fluidity of their phenotype. In this study, we employed the widely used M1/M2 classification for both macrophages and MDM, based on CD163 expression, as it is commonly applied in the literature to categorize macrophages [49–51].

Our study reveals elevated numbers of MDM within the TME in patients with melanoma, who respond to anti-PD-1 treatment. This suggests that the influx of proinflammatory circulating monocytes, capable of differentiating into (proinflammatory) macrophages in the TME, holds significance for the response to ICI. Indeed, RNAseq analyses confirmed the association of MDM presence in the TME with therapy response, PFS and OS. Furthermore, our analyses revealed that the expression of several chemokines, including CSF1, CSF1R, CCL2, CCL5 and CCR7, correlates with an MDM^HIGH^ gene signature, suggesting their role in attracting MDMs to the TME.

Non-responders showed increased numbers of CD16^-^ M2 macrophages with low immune checkpoint expression, co-localizing with T-cells within the TME. Studies on primary cutaneous melanoma show that M2-like macrophages are associated with poor prognosis and poor ICI response by inhibiting T-cell activation [52–54]. In addition, co-localization of macrophages and CD8^+^ T-cells in the tumor stroma has been implicated in limiting T-cell motility and reducing ICI response in lung squamous cell carcinoma [55]. Together, M2 macrophage’s co-localization with T-cells seem to contribute to ICI resistance in metastatic melanoma.

Currently, the available data regarding the role of macrophage subsets in metastatic melanoma remains limited. Previous studies showed that high CD16 expression, as well as intertumoral CD16^+^ macrophages levels, are associated with immune-rich tumors and subsequent better prognosis in metastatic melanoma patients treated with anti-PD-1 monotherapy, or in combination with anti-CTLA-4 [48]. However, these studies did not discriminate between pro-inflammatory (M1) and suppressive (M2) macrophages. In agreement with our study, others have reported an association between M2 macrophages in primary cutaneous melanoma and poor clinical outcome [52–54].

Our T-cell analyses demonstrated that overall, responders had increased numbers of infiltrating T-cells, especially CD4^+^ CTLs, CD8^+^ CTLs, CD8^+^ T_MEM_, CD8^+^ TPEX and CD8^+^ TEX, although there was high inter-patient variability. Other studies also implied that these CD8^+^ TPEX are crucial for immunotherapy response in metastatic melanoma [46, 56]. In accordance with other studies, CD8^+^ TPEX [10, 46], as well as CD8^+^ CTLs [57] in metastatic melanoma patients treated with ICI, are associated with a positive outcome. Furthermore, circulating CD8^+^ TMEMs were associated with durable responses in metastatic melanoma [58]. An in-depth evaluation of the exhausted T-cell subsets revealed that we were able to detect different CD8^+^ T-cell exhaustion states in human melanoma based on Ly108, CD69, TOX, and Tcf1/7, which were previously described in the murine melanoma model [38]. CD4^+^ PTEX and TEX could be distinguished based on Tcf1/7 and TOX, respectively. Lastly, we observed elevated numbers of infiltrating CD4^+^ CTLs in pre-anti-PD-1 treatment biopsies of metastatic melanoma patients, suggesting that CD4 T-cells may contribute actively to an anti-tumor response. This aligns with studies in other cancer types showing that CD4^+^ CTLs have a positive predictive outcome [43, 44].

In our study, we found elevated levels of CXCL9, CXCL10 and CXCL13, which promote T-cell recruitment into the TME, in ICI responders. As implicated by previous studies, chemotaxis of T-cells is important for ICI response but not sufficient [56, 59–62]. Here, we show that promoting the influx of both T-cells and monocytes can be relevant for successful immune therapy in melanoma, and that the myeloid cells in the TME produce T cell- attracting chemokines and vice-versa. Relevant chemokines and receptors for myeloid recruitment and differentiation such as CSF1, CCL5 and CCR7 were increased in ICI responders with MDM^high^ gene signatures. Drugs targeting these chemokines or their receptors have shown promise in preclinical and clinical settings in other tumor types [47]. While blockade of myeloid trafficking may have a beneficial effect by preventing influx of suppressive myeloid subsets, it will simultaneously inhibit influx of MDMs and other, inflammatory, myeloid cells that can be relevant for ICI response. For example, we demonstrate that myeloid-related chemokines, such as CCL5, are not only crucial for recruiting M2 macrophages but also for the infiltration of MDMs. The infiltration of these subsets correlates with ICI response in metastatic melanoma, therefore addition of CCL5 blockade to current ICI strategies could impact the ICI response negatively.

Moreover, our results highlight that in responders PD-1, PD-L1, VISTA, SIRPα, and CD47 are highly expressed. We demonstrated that both myeloid and T cells with high immune checkpoint expression were significantly closer to tumor cells than their immune checkpoint-low counterparts. Given that immune checkpoint interactions are known to induce functional inactivation of myeloid and T cells [63], this spatial association suggests that immune cells in closer proximity to tumor cells may be less active and, consequently, less effective in inducing an anti-tumor response. The underlying mechanisms driving this phenomenon remain unclear, but one plausible explanation is that tumor cells actively shape their microenvironment by secreting immunosuppressive cytokines and chemokines [64, 65], leading to the upregulation of immune checkpoints on nearby immune cells. Further investigation should elucidate the precise molecular pathways involved in this process.

For the myeloid checkpoints VISTA and SIRPα, we show that elevated expression is correlated with response. By contrast, in other VISTA and SIRPα studies expression was related to poor prognosis [66, 67], and VISTA expression was linked to anti-PD-1 resistance in melanoma [68, 69]. Myeloid-targeting ICI strategies, such as such as CD47-SIRPα and VISTA blockade, are currently tested in clinical trials [70, 71]. Given that high expression of checkpoints PD-1, PD-L1, VISTA, SIRPα, and CD47 expression associated with anti-PD-1 response in our cohort, it remains to be investigated whether combination therapy with myeloid checkpoint blockade can overcome anti-PD-1 resistance in metastatic melanoma.

Together, our results implicate that the influx of MDM with high checkpoint expression and T-cell subsets in the TME distinguishes metastatic melanoma with a favorable response to anti-PD-1 treatment and improved survival. To optimize ICI response in melanoma, we propose a combination approach, integrating existing T-cell targeting ICI strategies and recruitment of T-cells and monocytes combined with metabolic reprogramming of infiltrated monocytes and MDMs towards an inflammatory M1 phenotype [72]. Optimal recruitment and activation of both myeloid and T-cells into the TME may enhance the success of immune therapy in cancer and treatment efficacy for patients with metastatic melanoma.

## Data and materials availability

Analysis was performed in R (Version 4.2.2) using publicly available packages. All data and scripts are publicly available in a Zenodo repository (10.5281/zenodo.15703052) and Github (https://github.com/VercoulenLab/Melanoma-BJC-2025).

## Supplementary information

Supplementary Tables

Supplementary Figures
