# Supplementary material for: Anti-PD-1 treatment response is associated with the influx of circulating myeloid and T-cell subsets into the metastatic melanoma tumor microenvironment
Source: Br J Cancer. 2025 Sep 2;133(9):1250–64. doi: 10.1038/s41416-025-03137-8 (PMC12572286; doi:10.1038/s41416-025-03137-8)
Supplement: Supplementary file 1 — Supplementary Tables [file 41416_2025_3137_MOESM1_ESM.pdf]

Supplementary Tables

Supplementary Table 1. Characteristics of anti-PD-1 responding and non-responding patients

	Responders	Non-Responders
Age (yr), median IQR	58 (57-61)	67.5 (49.5-71.75)
Sex (male), N (%)	4 (57)	7 (87.5)
Driving mutation		
BRAF, N (%)	4 (57)	3 (37.5)
NRAS, N (%)	2 (29)	3 (37.5)
None, N (%)	1 (14)	2 (25)
Anti-PD-1 Therapy		
Nivolumab, N (%)	1 (14)	3 (37.5)
Pembrolizumab, N (%)	6 (86)	5 (62.5)
OS (months), median (IQR)	70 (60.5-74)	18.5 (11-24)
PFS (months), median (IQR)	70 (60.5-74)	2.75 (3-3)

Supplementary Table 2. Baseline patient characteristics.

**Patient**	**Treatment**	**Response**	**RECIST**	**Driving mutation (BRAF/NRAS)**	**Tumor side**	**Sample type**
**1**	Pembrolizumab	Responder	CR	NRAS	Lymph node	TR
**2**	Pembrolizumab	Responder	CR	None	Lung	TR
**3**	Pembrolizumab	Responder	PR	BRAF	Lymph node	NB
**4**	Pembrolizumab	Responder	CR	BRAF	Lymph node	NB
**5**	Pembrolizumab	Responder	CR	NRAS	Lymph node	NB
**6**	Pembrolizumab	Responder	CR	BRAF	Soft tissue	NB
**7**	Nivolumab	Responder	PR	BRAF	Lymph node	NB
**8**	Nivolumab	Non-responder	PD	BRAF	Skin/lymph node	NB
**9**	Pembrolizumab	Non-responder	PD	NRAS	Subcutaneous	NB
**10**	Pembrolizumab	Non-responder	PD	None	Skin/lymph node	TR
**11**	Pembrolizumab	Non-responder	PD	None	Liver	TR
**12**	Pembrolizumab	Non-responder	PD	NRAS	Skin	TR
**12**	Pembrolizumab	Non-responder	PD	NRAS	Lymph node	TR
**13**	Nivolumab	Non-responder	PD	NRAS	Lymph node	TR
**14**	Nivolumab	Non-responder	PD	BRAF	Lymph node	TR
**15**	Pembrolizumab	Non-responder	PD	BRAF	Lymph node	TR
**15**	Pembrolizumab	Non-responder	PD	BRAF	Lymph node	TR
Abbreviations: CR: complete response, NB: Needle Biopsy, PD: progressive disease, PR: partial response, TR: Tissue Resection**.**						

Supplementary Table 3. Myeloid Antibody Panel for Imaging Mass Cytometry.

Target Name	Isotope	Clone	Supplier	Cat #	Dilution
**CD10**	140Ce	E5P7S	Cell Signaling Technology	54370SF	1:50
**CD56**	141Pr	NCAM1/784	Abcam	ab216010	1:100
**MelanA**	142Nd	Polyclonal	Dako	A045229-2	1:50
**MPO**	143Nd	E1E7I	Cell Signaling Technology	CST13917BF	1:100
**CD16**	144Nd	EPR22409-124	Abcam	ab252908	1:50
**CD33**	145Nd	Polyclonal	Fluidigm	3145017D	1:75
**CD15**	146Nd	W6D3	Biolegend	323002	1:200
**CD163**	147Sm	D6U1J	Cell Signaling Technology	25121SF	1:200
**PD-L1**	148Nd	E1L3N	Cell Signaling Technology	CST13684BF	1:50
**SOX10**	149Sm	EPR4007	Abcam	ab220078	1:100
**CD47**	150Nd	EPR21794	Abcam	ab233122	1:100
**IFNγ**	151Eu	D3H2	Cell Signaling Technology	CST8455BF	1:100
**iNOS**	152Sm	SP126	Abcam	ab239990	1:50
**NFkB**	153Eu	L8F6	Cell Signaling Technology	64921SF	1:200
**CD68**	154Sm	KP1	Biolegend	916104	1:200
**FOXP3**	155Gd	236A/E7	Abcam	ab96048	1:50
**CD4**	156Gd	EPR6855	Abcam	ab181724	1:50
**pSTAT3 (Tyr705)**	158Gd	D3A7	Cell Signaling Technology	73533SF	1:200
**CD20**	159Tb	H1	BD Biosciences	555677	1:200
**IL-10**	160Gd	Polyclonal	R&D	AF-217-NA	1:200
**SIRPa**	-	D6I3M	Cell Signaling Technology	47027SF	1:100
**LOX-1**	162Dy	Polyclonal	Invitrogen	PA5-80872	1:100
**CD123**	163Dy	IL3RA/1531	LsBio	LS-C782564	1:100
**CD45**	164Dy	D9M8I	Cell Signaling Technology	CST13917BF	1:200
**PD-1**	165Ho	EPR4877(2)	Abcam	ab186928	1:50
**VISTA**	166Er	D1L2G	Cell Signaling Technology	82119SF	1:100
**Arginase-1**	167Er	SI6arg	eBioscience	14-9779-82	1:400
**Ki-67**	168Er	B56	BD	550609	1:200
**Granzyme B**	169Tm	D6E9W	Cell Signaling Technology	CST46890BF	1:200
**CD3**	170Er	Polyclonal	DAKO	A045229-2	1:100
**pERK (Thr202/Tyr204)**	171Yb	D13.14.4E	Cell Signaling Technology	CST4370BF	1:100
**CD8a**	172Yb	C8/144B	Thermo Fisher Scientific	14-0085-82	1:200
**HLA-DRDPDQ**	173Yb	TAL 1B5	Abcam	Ab176408	1:800
**CD14**	174Yb	EPR3653	Abcam	ab226121	1:200
**CD11b**	175Lu	D6X1N	Cell Signaling Technology	CST49420BF	1:200
**Histone H3**	176Yb	D1H2	Cell Signaling Technology	CST4499BF	1:800
**Intercalator**	191Ir; 193Ir	-	Fluidigm	201192B	1:500
**Goat-anti-Rabbit Highly Cross Absorbed**	161Sm	Polyclonal	Invitrogen	A16112	1:100

Supplementary Table 4. T-cell Antibody Panel for Imaging Mass Cytometry.

Target Name	Isotope	Clone	Supplier	Cat #	Dilution
**CD56**	141Pr	NCAM1/784	Abcam	ab216010	1:100
**MelanA**	142Nd	Polyclonal	Dako	A045229-2	1:50
**CD45**	143Nd	D9M8I	Cell Signaling Technology	CST13917BF	1:100
**CD11c**	144Nd	EP1347Y	Abcam	ab216655	1:100
**MHC-I**	145Nd	F-3	Santa-Cruz	sc-55582	1:100
**CD16**	146Nd	EPR16784	Abcam	ab256582	1:100
**non-phospho (active) beta catenin**	147Sm	D13A1	Cell Signaling Technology	CST8814BF	1:50
**PD-L1**	148Nd	E1L3N	Cell Signaling Technology	CST13684BF	1:50
**SOX10**	149Sm	EPR4007	Abcam	ab220078	1:100
**CD68**	150Nd	KP1	Biolegend	916104	1:400
**IFNγ**	151Eu	D3H2	Cell Signaling Technology	CST8455BF	1:100
**CD45RO**	152Sm	UCHL1	Cell Signaling Technology	CST55618BF	1:100
**TOX**	153Eu	E6I3Q	Cell Signaling Technology	CST73756	1:50
**CTLA-4**	-	CAL49	Abcam	ab237712	1:100
**FOXP3**	155Gd	236A/E7	Abcam	ab96048	1:50
**CD4**	156Gd	EPR6855	Abcam	ab181724	1:100
**CD103**	157Gd	EPR4166(2)	Abcam	ab221210	1:100
**CD69**	158Gd	10803-1-AP	Proteintech		1:100
**CD19**	159Tb	60MP31	Invitrogen	14-0194-82	1:100
**Ly108**	160Gd	005	Sino	11945-R005	1:100
**CD138**	161Dy	MI15	Biolegend	356502	1:100
**CD8a**	162Dy	C8/144B	Thermo Fisher Scientific	14-0085-82	1:100
**BCL6**	163Dy				1:50
**pS6 (Ser235/236)**	164Dy	D57.2.2E	Cell Signaling Technology	CST4858BF	1:200
**PD-1**	165Ho	EPR4877(2)	Abcam	ab186928	1:50
**CD103**	166Er	EPR4166(2)	Abcam	ab221210	1:50
**Tbet**	167Er	D6N8B	Cell Signaling Technology	CST13232	1:50
**Ki-67**	168Er	B56	BD	550609	1:100
**Granzyme B**	169Tm	D6E9W	Cell Signaling Technology	CST46890BF	1:100
**CD3**	170Er	Polyclonal	DAKO	A045229-2	1:100
**pERK (Thr202/Tyr204)**	171Yb	D13.14.4E	Cell Signaling Technology	CST4370BF	1:100
**TCF1**	172Yb	C63D9	Cell Signaling Technology	CST2203BF	1:50
**HLA-DRDPDQ**	173Yb	CR3/43	Abcam	ab7856	1:100
**CD39**	174Yb	A1	Biolegend	328202	1:50
**pAKT (Ser473)**	175Lu	D9E	Cell Signaling Technology	CST4060BF	1:100
**Histone H3**	176Yb	D1H2	Cell Signaling Technology	CST4499BF	1:000
**Intercalator**	191Ir; 193Ir	-	Fluidigm/ Standard Biotools	201192B	1:500
**Goat-anti-Rabbit Highly Cross Absorbed**	154Sm	Polyclonal	Invitrogen	A16112	1:100

Supplementary Table 5. Boolean Rules for identification of immune cell subsets

Candidate Cell Type	Marker Expression
CD4$^+$ naive T-cell	CD45$^+$ **CD3$^+$** **CD4$^+$** CD8$^-$ CD45RO$^-$ CD103$^-$GranzymeB$^-$ TOX$^-$ Tcf1/7$^-$ Tbet$^-$ FOXP3$^-$
CD8$^+$ naive T-cell	CD45$^+$ **CD3$^+$** CD4$^-$ **CD8$^+$** CD45RO$^-$ CD103$^-$ GranzymeB$^-$ TOX$^-$ Tcf1/7$^-$ Tbet$^-$ FOXP3$^-$
T helper 1 cell	CD45$^+$ CD3$^+$ CD4$^+$ CD8$^-$ **Tbet$^+$** FOXP3$^-$
CD4$^+$ progenitor dysfunctional T-cell	CD45$^+$ CD3$^+$ CD4$^+$ CD8$^-$ TOX- **Tcf1/7+** FOXP3$^-$
CD4$^+$ terminal dysfunctional T-cell	CD45$^+$ CD3$^+$ CD4$^+$ CD8$^-$ **TOX$^+$** Tcf1/7$^-$ FOXP3$^-$
CD8$^+$ progenitor dysfunctional T-cell	CD45$^+$ CD3$^+$ CD4$^-$ CD8$^+$ TOX- **Tcf1/7+** FOXP3$^-$
CD8$^+$ terminal dysfunctional T-cell	CD45$^+$ CD3$^+$ CD4$^-$ CD8$^+$ **TOX$^+$** Tcf1/7$^-$ FOXP3$^-$
CD4$^+$ cytotoxic T-cell	CD45$^+$ CD3$^+$ CD4$^+$ CD8$^-$ **GranzymeB$^+$** FOXP3$^-$
CD8$^+$ cytotoxic T-cell	CD45$^+$ CD3$^+$ CD4$^-$ CD8$^+$ **GranzymeB$^+$** FOXP3$^-$
CD4$^+$ memory T-cell	CD45$^+$ CD3$^+$ CD4$^+$ CD8$^-$ **CD45RO$^+$** FOXP3$^-$
CD8$^+$ memory T-cell	CD45$^+$ CD3$^+$ CD4$^-$ CD8$^+$ **CD45RO$^+$** CD103$^-$ FOXP3$^-$
CD8$^+$ tissue resident memory T-cell	CD45$^+$ CD3$^+$ CD4$^-$ CD8$^+$ CD45RO$^+$ **CD103$^+$** TOX$^-$ Tcf1/7$^-$ Tbet$^-$ FOXP3$^-$
CD4$^+$ regulatory T-cell	CD45$^+$ CD3$^+$ CD4$^+$ CD8$^-$ **FOXP3$^+$**
CD8$^+$ regulatory T-cell	CD45$^+$ CD3$^+$ CD4$^-$ CD8$^+$ **FOXP3$^+$**
B cell	CD45$^+$ CD3$^-$ **CD19$^+$**
NK cell	CD45$^+$ CD3$^-$ **CD56$^+$** CD16$^+$
NKT cell	CD45$^+$ CD3$^+$ **CD56$^+$** CD16$^+$
Macrophage	**CD68$^+$** CD11c$^-$
Dendritic Cell	CD68$^-$ CD3$^-$ **CD11c$^+$** CD19$^-$
Other Immune Cell	**CD45$^+$** CD3$^-$ CD4$^-$ CD8$^-$ CD19$^-$ CD45RO$^-$ CD16$^-$ CD56$^-$ CD11c$^-$ CD68$^-$ GranzymeB$^-$ FOXP3$^-$ TOX$^-$ Tcf1/7$^-$ Tbet$^-$
Non-classified	**CD45$^-$ CD45RO$^-$**
Markers in **bold** represent the key markers used for lineage ranking.	

Supplementary Table 6. Overview of regions and analyzed cells regarding IMC panels.

		Myeloid Panel			T-Cell Panel		
**Patient**	**Tumor side**	**ROI (N)**	**Area (mm^2)**	**Analyzed Cells (N)**	**ROI (N)**	**Area (mm^2)**	**Analyzed Cells (N)**
**1**	Lymph node	3	0.75	4677	3	0.75	8403
**2**	Lung	3	0.75	5296	2	0.5	3899
**3**	Lymph node	3	0.75	8360	3	0.75	8672
**4**	Lymph node	2	0.50	3548	2	0.5	3072
**5**	Lymph node	3	0.75	8404	3	0.75	10609
**6**	Soft tissue	3	0.75	7876	3	0.75	15975
**7**	Lymph node	3	0.75	4700	3	0.75	13315
**9**	Subcutaneous	3	0.75	5486	3	0.75	14308
**10**	Skin/lymph node	3	0.75	6326	3	0.75	16178
**11**	Liver	3	0.75	5157	3	0.75	4921
**12**	Skin	3	0.75	3118	3	0.75	2917
**12**	Lymph node	3	0.75	4091	2	0.5	2935
**13**	Lymph node	3	0.75	5656	3	0.75	3024
**14**	Lymph node	3	0.75	3699	3	0.75	3152
**15**	Lymph node	3	0.75	3145	3	0.75	3011
**15**	Lymph node	3	0.75	4483	3	0.75	4904
Abbreviations: ROI: region of interest.							
