# Supplementary material for: Anti-PD-1 treatment response is associated with the influx of circulating myeloid and T-cell subsets into the metastatic melanoma tumor microenvironment
Source: Br J Cancer. 2025 Sep 2;133(9):1250–64. doi: 10.1038/s41416-025-03137-8 (PMC12572286; doi:10.1038/s41416-025-03137-8)
Supplement: Supplementary file 2 — Supplementary Figures [file 41416_2025_3137_MOESM2_ESM.pdf]

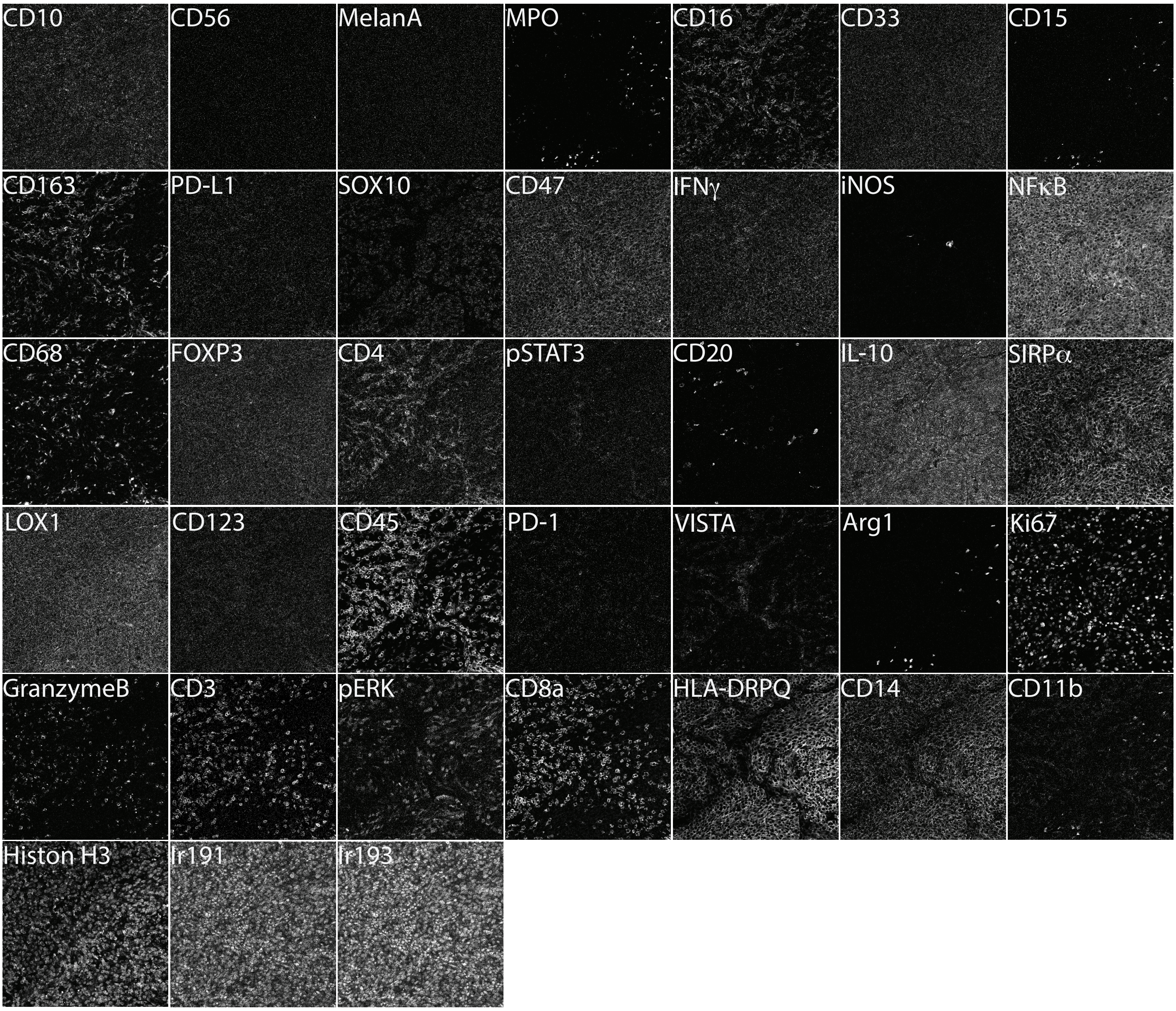

Supplementary Figure 1: Myeloid antibody panel on melanoma tumor tissue.
Representative images are shown per channel detected with the myeloid antibody panel.

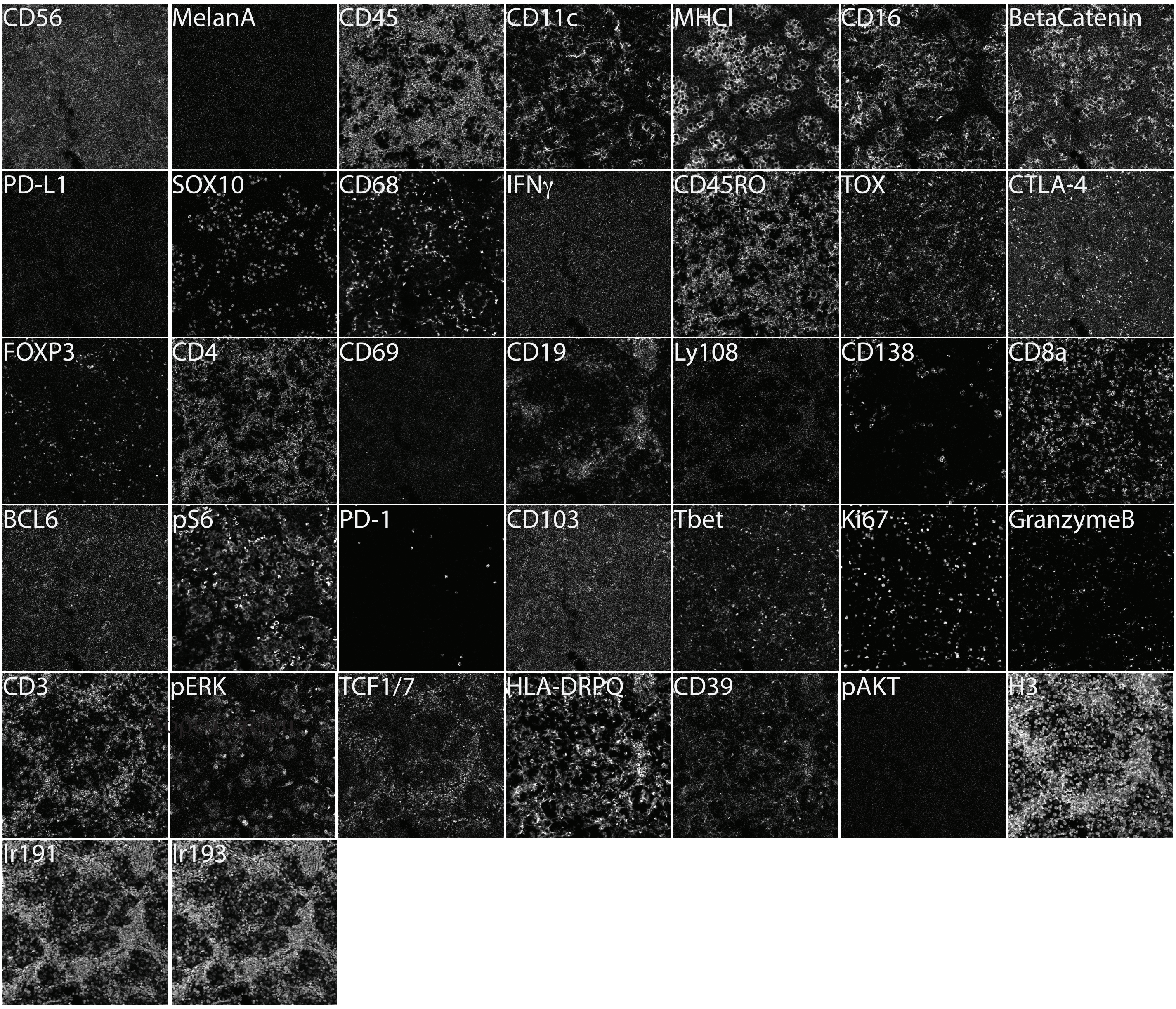

Supplementary Figure 2: T-cell antibody panel on melanoma tumor tissue.
Representative images are shown per channel detected with the T-cell antibody panel.

A.

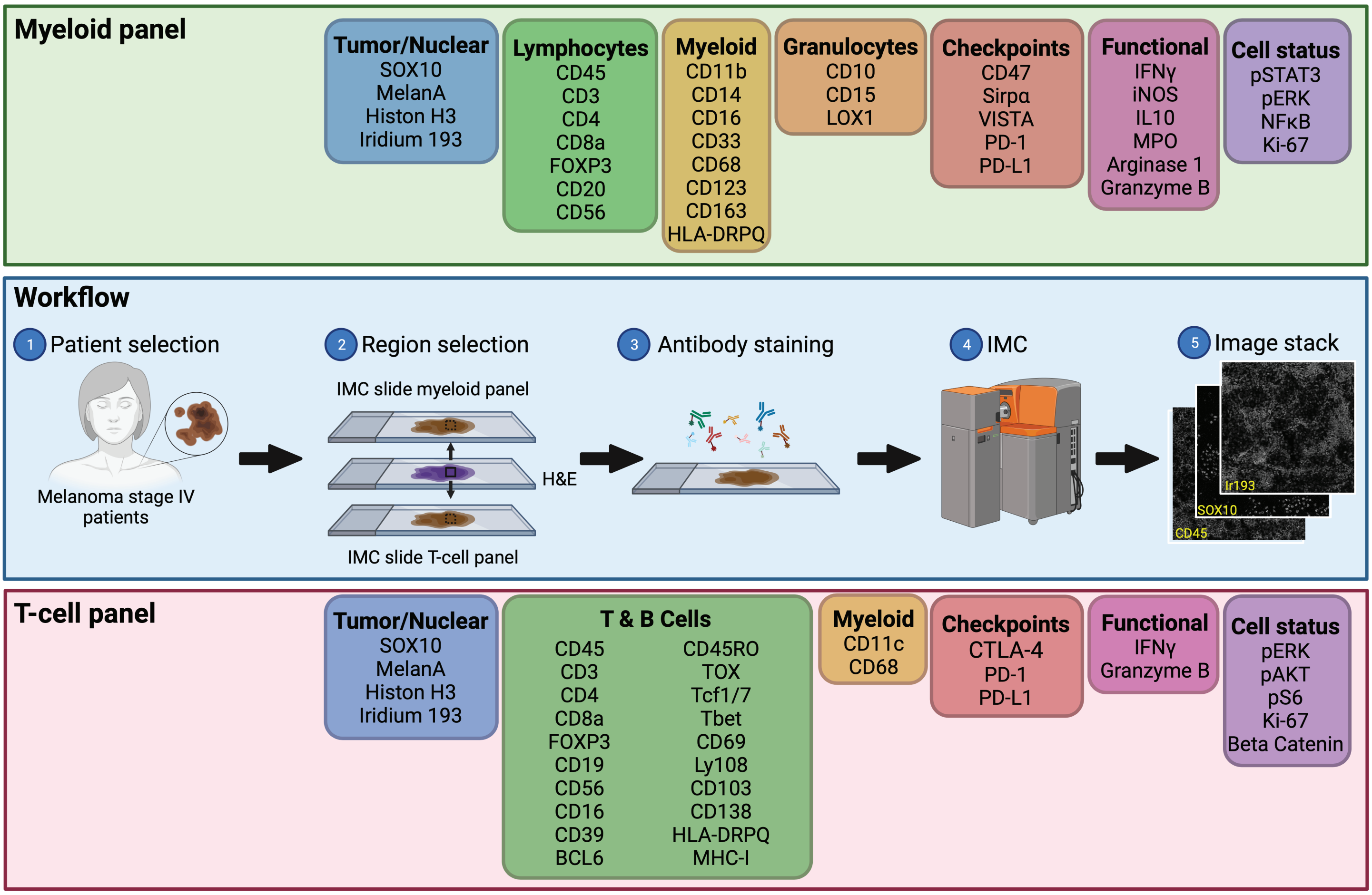

B.

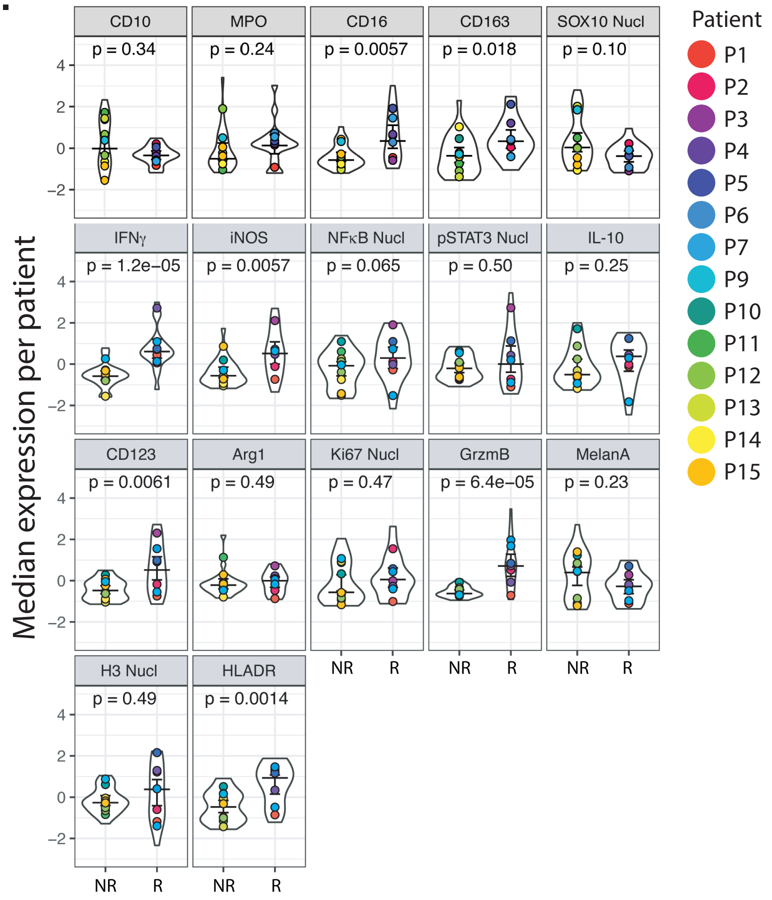

Supplementary Figure 3: Higher expression of immune-related markers in responding compared to non-responding metastatic melanoma patients. **A.** Illustration of the data acquisition workflow used for IMC: (1) tumor tissue derived from metastatic melanoma tissue was obtained prior to the start of anti-PD-1 treatment. (2) Three 500x500 mm regions of interest (ROI) were selected based on H&E-stained sections by a pathologist and used on sequential tissue sections for IMC stainings. (3) Tissue sections were stained with a myeloid (green) or T-cell (red) panel consisting of metal isotope-labeled antibodies. (4) ROI was ablated with a high-energy laser using IMC. (5) Each antibody resulted in a single image per sample with the metal isotope composition per pixel, together constructing a multi-channel image stack.
B. Violin plot with a median intensity of CD10, MPO, CD16, CD163, SOX10, IFNg, iNOS, NFkB, pSTAT3, IL-10, CD123, Arg1, KI67, Granzyme B (GrzmB), MelanA, H3 and HLA-DRDPDQ (HLADR) per patient split for therapy response. Patient ID is indicated in colors. Mann-Whitney U-test with Benjamini-Hochberg correction was used to calculate the statistical difference between non-responders and responders. Bars indicate the median with a 95% confidence interval.

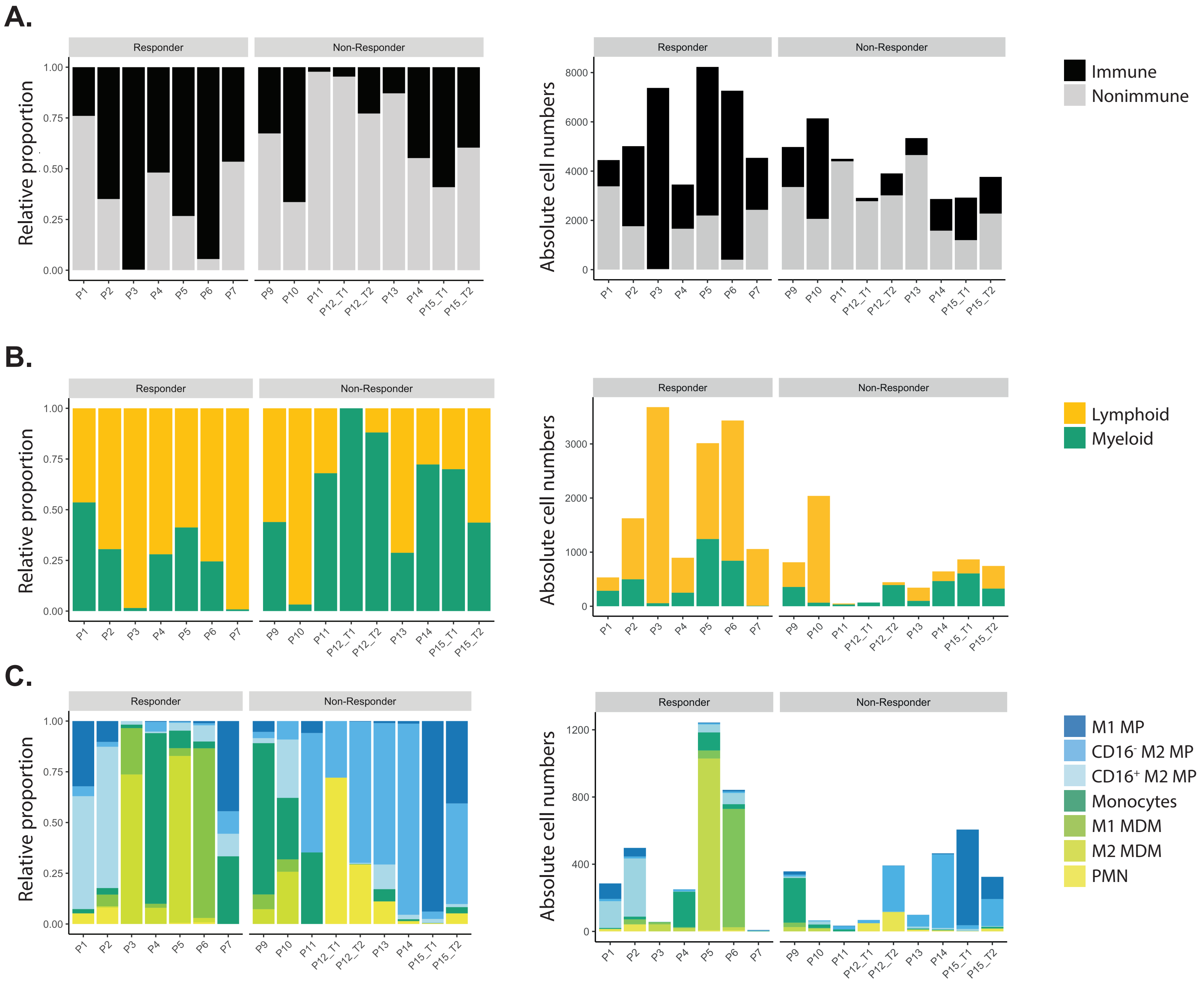

Supplementary Figure 4: Patient heterogeneity of immune cell clusters in metastatic melanoma.
A. Stacked bar plot representing the proportions (left) and total abundance (right) of annotated immune versus non-immune cell types stratified per patient. **B.** Stacked bar plot representing the proportions (left) and total abundance (right) of annotated myeloid versus lymphoid cell types stratified per patient. **C.** Stacked bar plot representing the proportions (left) and total abundance (right) of annotated myeloid cell types stratified per patient. For all patients, the abundance is shown as the mean of 3 ROIs (total 0.75mm2).

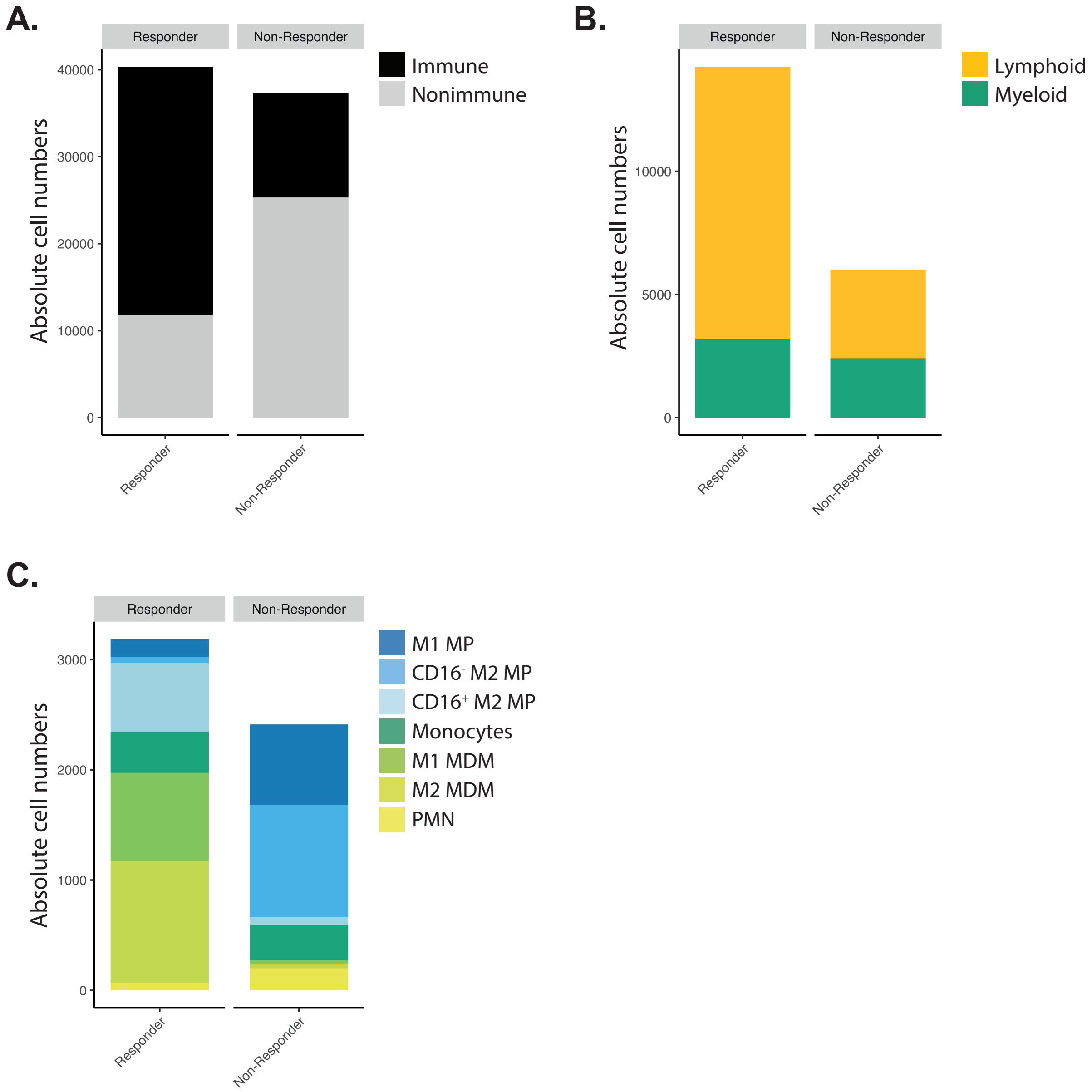

Supplementary Figure 5: Cell density of immune cell clusters in metastatic melanoma.
A. Stacked bar plot representing the total abundance of annotated immune versus non-immune cell types. **B.** Stacked bar plot representing the total abundance of annotated myeloid versus lymphoid cell types. **C.** Stacked bar plot representing the total abundance of annotated myeloid cell types. The mean abundance was first calculated for each individual patient (mean of 3 ROIs (total 0.75mm2)). Then, the mean abundance was calculated for all responding (left, n=7) and non-responding (right, n=7) patients combined.

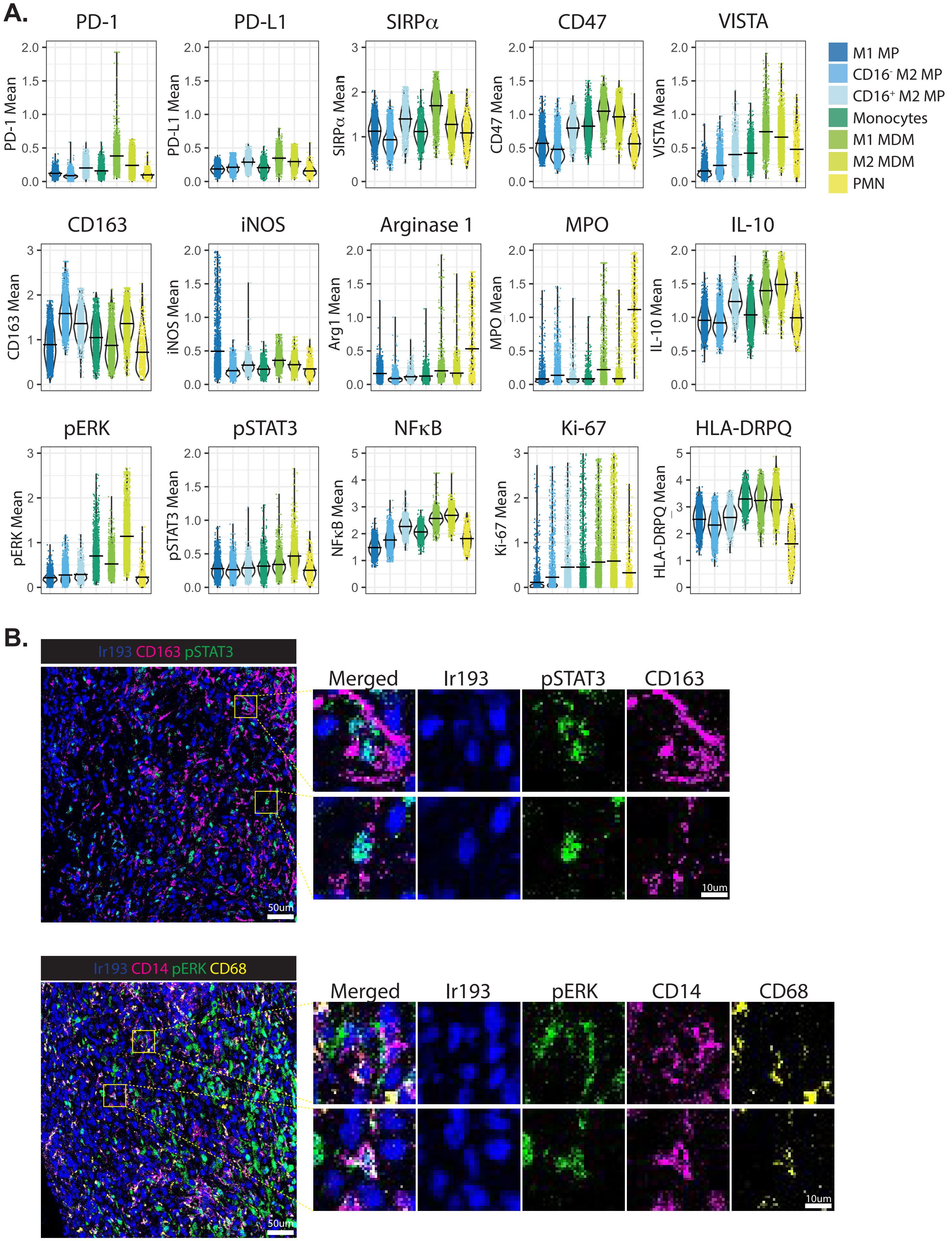

Supplementary Figure 6: Differences in marker expression between myeloid cell types.
A. Violin plots indicating the median expression of immune checkpoints, functional markers, and signaling markers in the identified myeloid cell clusters (M1 MP, CD16- M2 MP, CD16+ M2 MP, Monocytes, M1 MDM, M2 MDM, and PMN). Every dot is a single cell. Bars indicate the median with a 95% confidence interval. **B.** Representative IMC stainings showing pSTAT3 staining and pERK staining in MDMs. The scale bar is 10 mm.

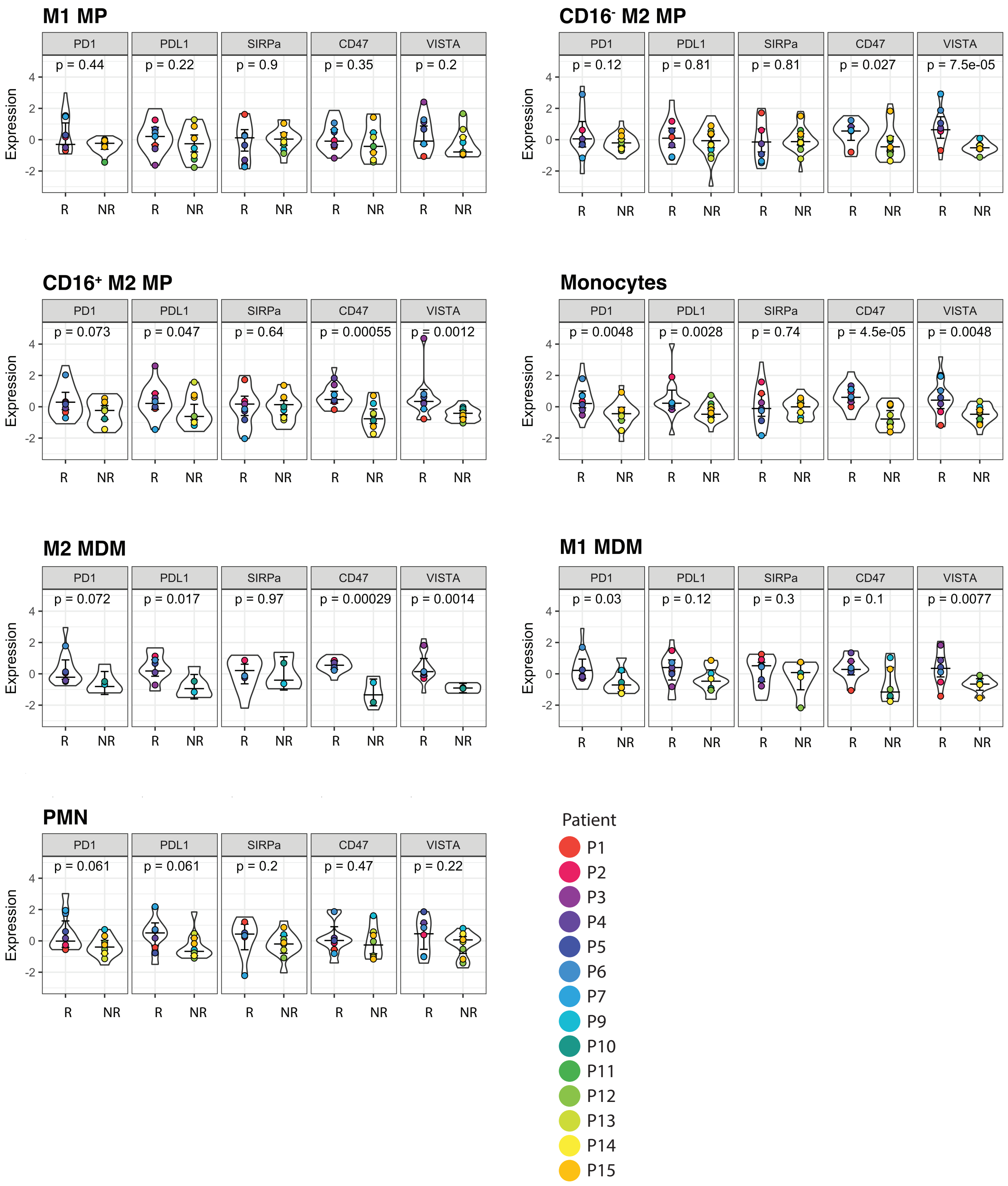

Supplementary Figure 7: Differences in checkpoint expression between myeloid cell types.
Violin plots indicating the median expression of immune checkpoints in the identified myeloid cell clusters (M1 MP, CD16- M2 MP, CD16+ M2 MP, Monocytes, M1 MDM, M2 MDM, and PMN) in responders (R) and non-responders (NR). Patient ID is indicated in colors. Mann-Whitney U-tests were used to calculate the statistical difference between responders and non-responders. Bars indicate the median with a 95% confidence interval.

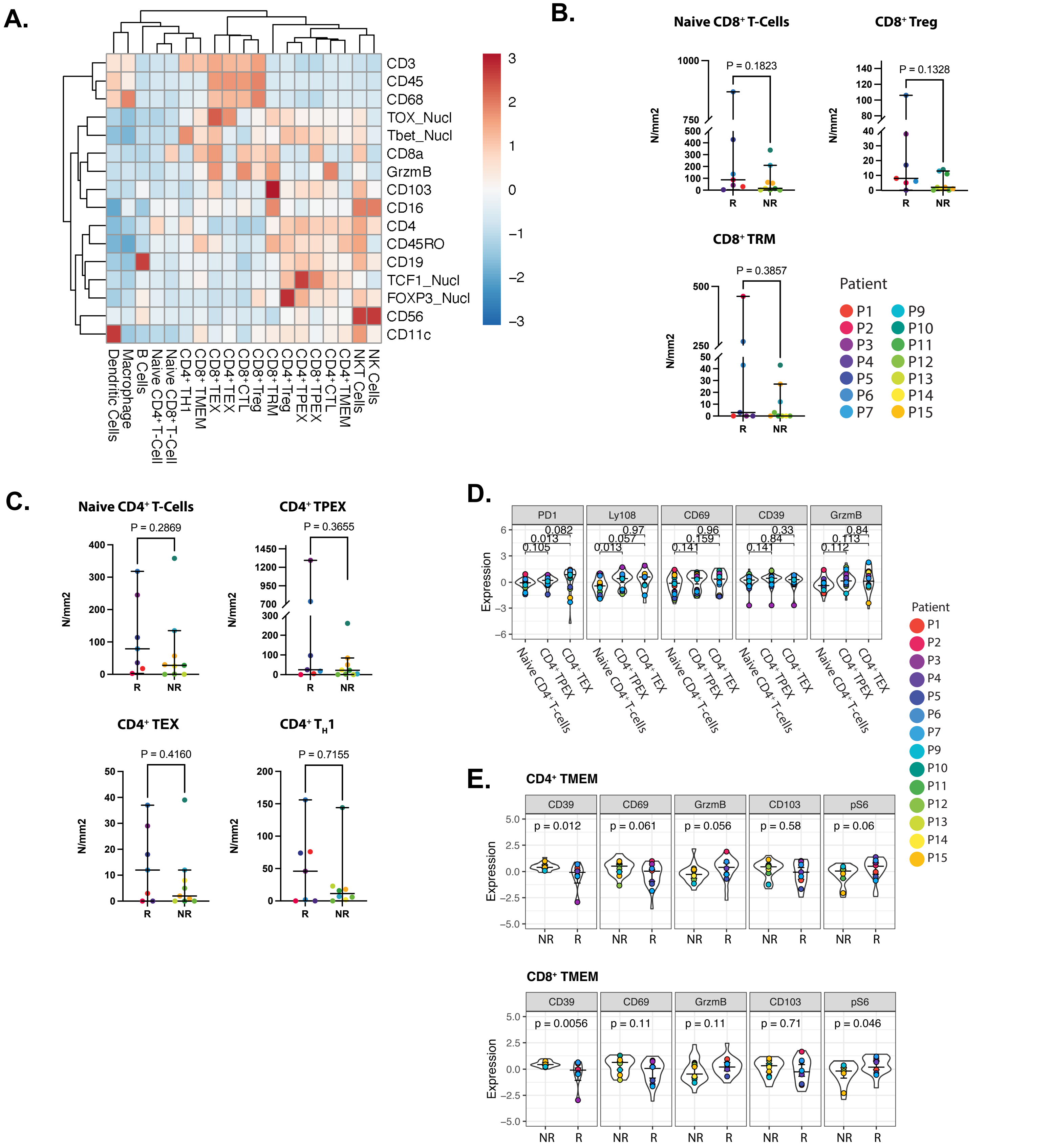

Supplementary Figure 8: Marker expression in CD4+ exhausted T-cell subsets and CD4+ and CD8+ TMEM.
A. Hierarchical clustering heatmap of the median marker intensity per patient indicated for the annotated T-cell types. **B.** Dot plots of the median abundancy of annotated CD8+ T-cells subsets including naive CD8+ T-cells, regulatory CD8+ T-cells (CD8+ Treg), CD8+ tissue resident T-cells (CD8+ TRM), stratified for responder (R) and non-responder (NR). Each dot is a patient. The bars indicate the median with a 95% confidence interval. Mann-Whitney U-test was used to calculate the statistical difference between non-responders and responders. **C.** Dot plots of the median abundancy of annotated CD4+ T-cells subsets, including naive CD4+ T-cells, T helper 1 cells (TH1), CD4+ progenitor exhausted T-cells (CD4+ TPEX), and CD4+ terminally exhausted T-cells (CD4+ TEX), stratified by responder (R) and non-responder (NR). Each dot is a patient. The bars indicate the median with a 95% confidence interval. Mann-Whitney U-test was used to calculate the statistical difference between non-responders and responders. **D.** Violin plots indicating the median expression of IFNg in CD4+ naive T-cells, CD8+ naive T-cells, CD4+ CTLs, CD8+ CTLs in responders (R) Patient ID is indicated in colors. Nonparametric T-tests were used to calculate the statistical difference between T-cell subsets. Bars indicate the median with a 95% confidence interval. **E.** Violin plot with the median intensity of PD1, Ly108, CD39, CD69, and Granzyme B (GrzmB) in CD4+ naive T-cells, CD4+ TPEX and CD4+ TEX cells. Patient ID is indicated in colors. Mann-Whitney U-test was with Benjamini-Hochberg correction was used to calculate the statistical difference between non-responders and responders. Bars indicate the median with a 95% confidence interval.

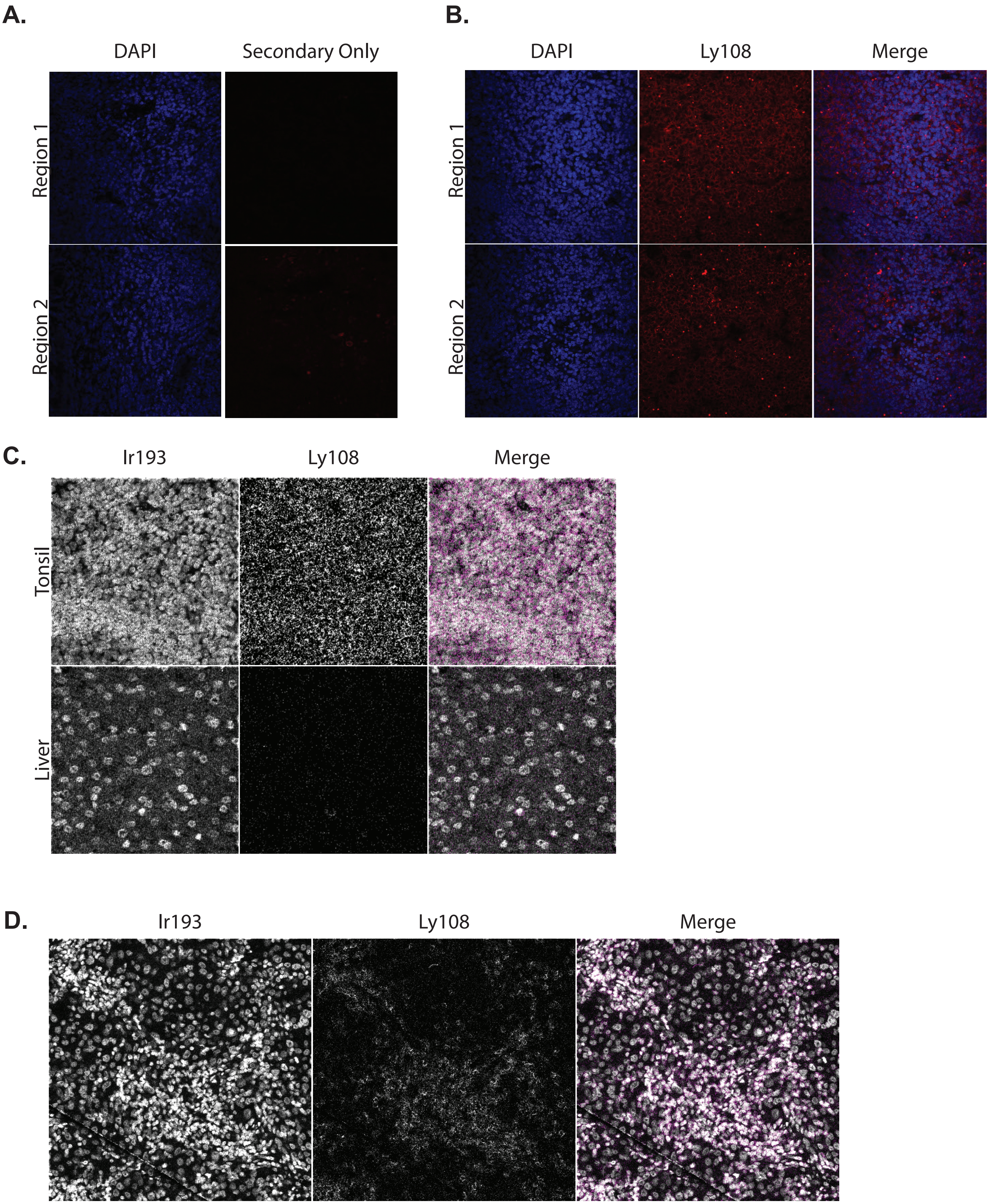

Supplementary Figure 9: Ly108 IF and IMC staining.
A. Negative control with secondary only staining on 2 regions in tonsil.
B. Ly108 IF staining (primary + Alexa 568 secondary) on tonsil.
C. IMC staining of the conjugated Ly108 antibody on tonsil (positive control) and liver (negative control).
D. IMC staining of Ly108 on melanoma tissue.

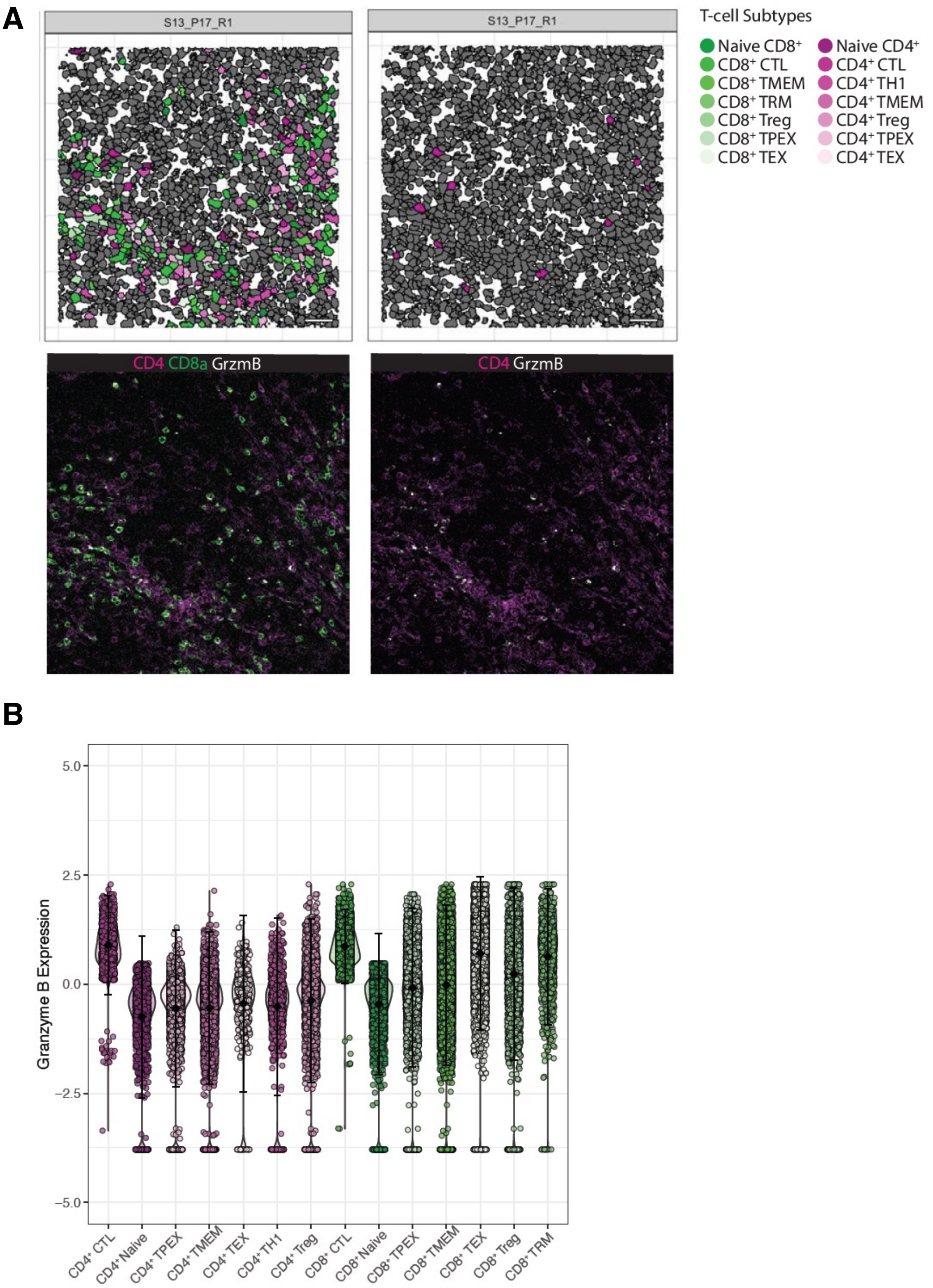

Supplementary Figure 10: CD4$^+$ CTLs.

A. Overlays and confetti plots illustrating the spatial distribution of CD4$^+$ and CD8$^+$ T-cell subsets expressing Granzyme B are shown for each ROI included in this study.

B. Distribution of Granzyme B expression in the different annotated T-cell subsets.

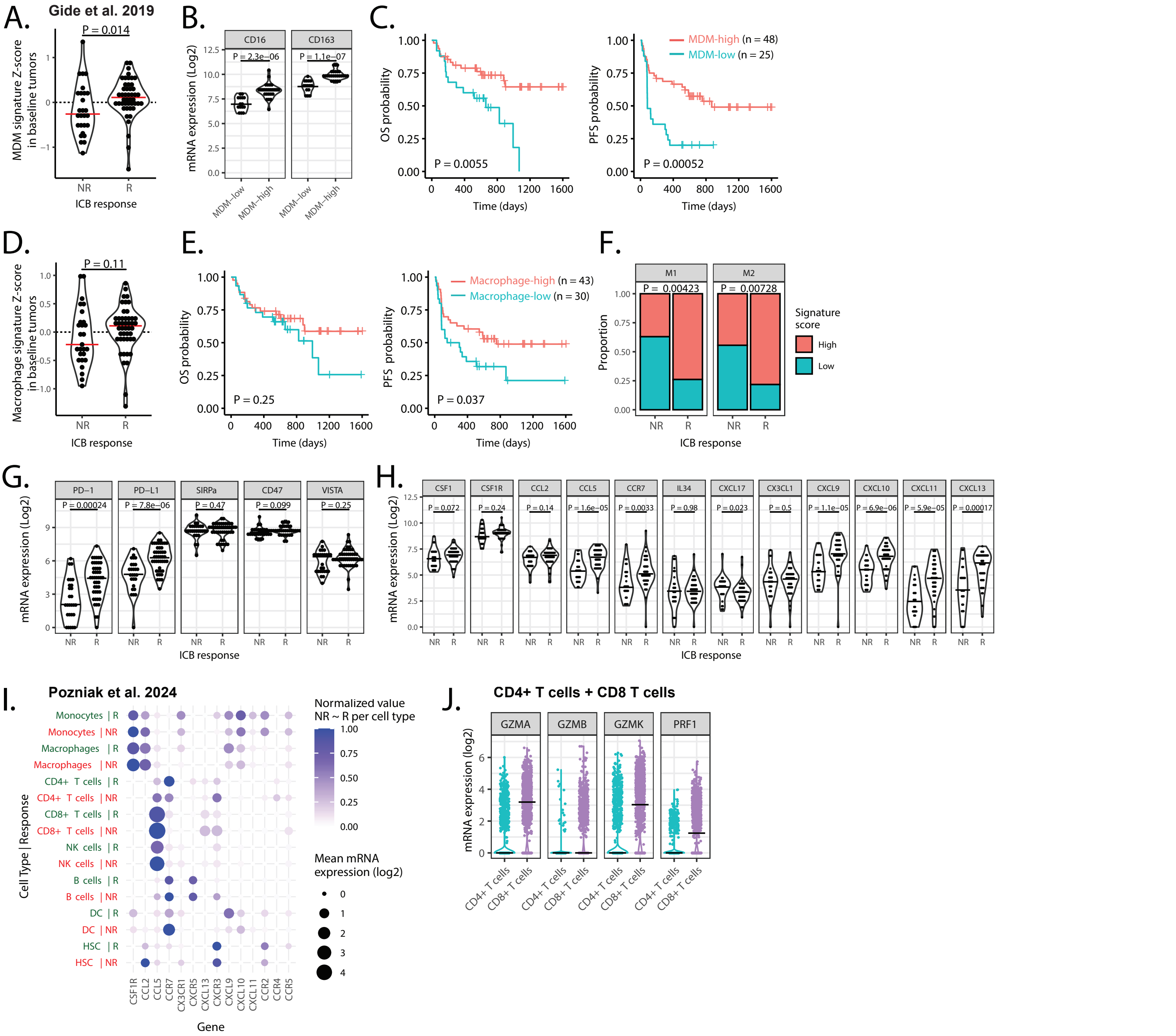

Supplementary Figure 11: Transcriptome analyses of (bulk) tumors and (single) immune cells prior ICI treatment. **A-C.** Analyses of bulk RNAseq data (*Gide et al. 2019*). **A.** Violin plots illustrating the Z-scores of the 'monocyte-derived-macrophage' (MDM) signature in tumors of non-responders (NR) and responders (R) to prior immune checkpoint inhibition (ICI) therapy, including mono anti-PD1 treatment or combined anti-PD1 and anti-CTLA4 treatment. Mann-Whitney U-test was applied. **B.** Violin plots illustrate the messenger RNA (mRNA) expression (log2-transformed RPM) of CD16 and CD163 in MDMLow and MDMHigh tumors of metastatic melanoma patients prior to ICI treatment. Mann-Whitney U-test was applied. **C.** Kaplan–Meier curves demonstrate overall survival (OS, left) and progression-free survival (PFS, right) in tumor subgroups characterized by the MDM signature using the k-means algorithm. Samples of metastatic melanoma patients were obtained prior to ICI treatment. A two-sided log-rank test was applied. **D.** Violin plots illustrating the Z-scores of the 'Macrophage' signature in tumors of non-responders (NR) and responders (R) prior to ICI treatment. Mann-Whitney U-test was applied. **E.** Kaplan–Meier curves demonstrate OS (left) and PFS (right) in tumor subgroups characterized by the 'Macrophage' signature using the k-means algorithm. Samples of metastatic melanoma patients were obtained prior to ICI treatment. A two-sided log-rank test was applied. **F.** Stacked bar plot illustrates the proportion of M1- and M2-high and low tumors in ICI non-responder (NR) and responder (R) patients. Samples of metastatic melanoma patients were obtained before ICI treatment. Chi-squared test was applied. **G.** Violin plots illustrate the mRNA expression (log2-transformed RPM) of immune checkpoint-related genes (PD-1 (PDCD1), PD-L1 (CD274), SIRPα (SIRPA), CD47 (CD47), and VISTA (VSIR) in ICI non-responder (NR) and responder (R) patients. Mann-Whitney U-test was applied. **H.** Violin plots illustrate the mRNA expression (log2-transformed RPM) of cytokine and chemokine genes (CSF1, CSF1R, CCL2, CCL5, CCR7, IL34, CXCL17, CX3CL1, CXCL9, CXCL10, CXCL11, CXCL13) in ICI non-responder (NR) and responder (R) patients. **I.** Dot plots illustrate gene expression across immune cell types and ICI responses (5 responders, 11 nonresponders) (*Pozniak et al. 2024*). Circle size reflects absolute mean mRNA expression (log2-transformed RPM), and fill color (white to blue) shows normalized expression between NR and R within each immune cell type. **J.** Violin plots illustrate the mRNA expression (log2-transformed RPM) of Granzyme A (GMZA), Granzyme B (GZMB), Granzyme K (GZMK), and Perforin1 (PRF1) in CD4+ T cells and CD8+ T cells.

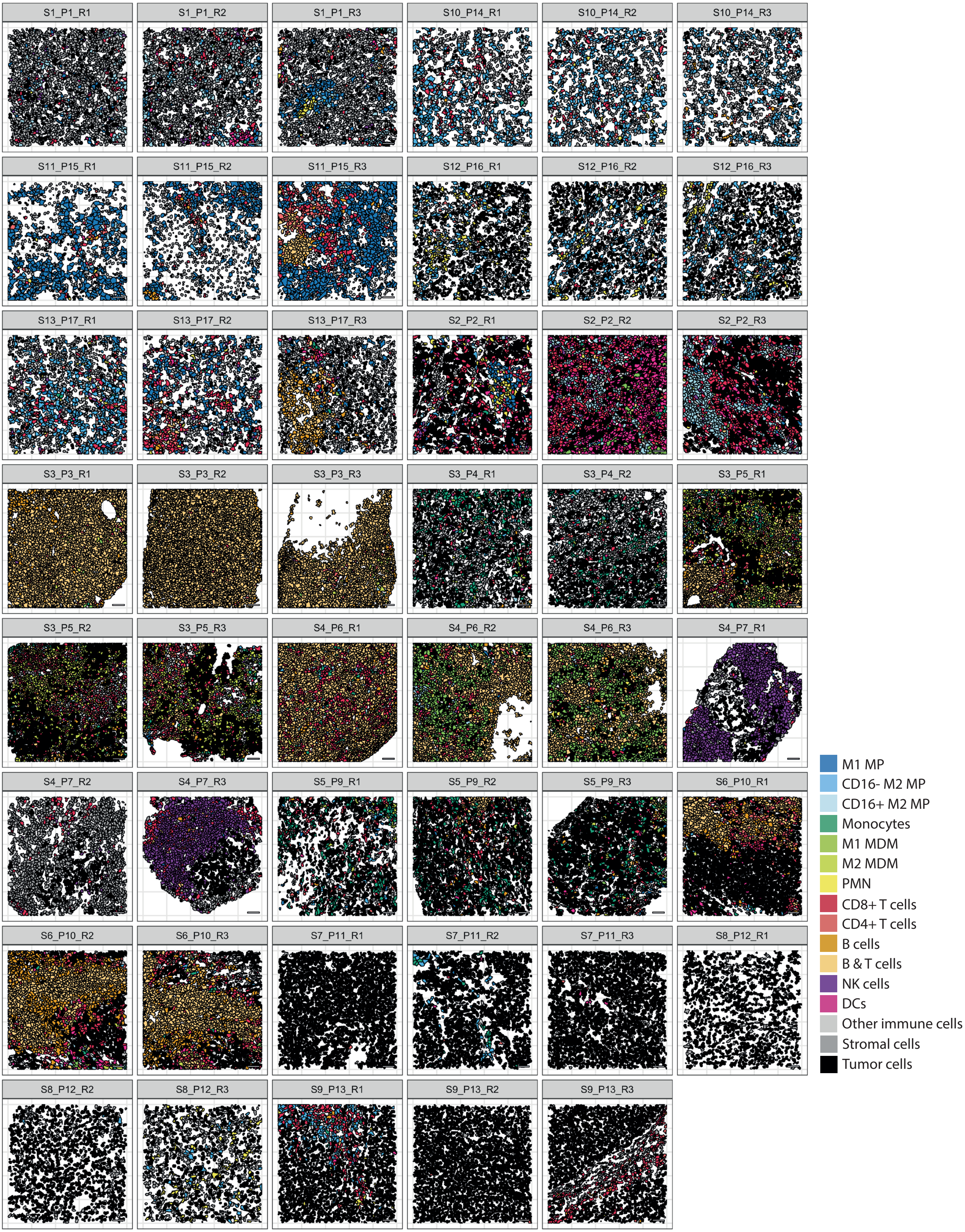

Supplementary Figure 12: Spatial distribution of cell subsets in metastatic melanoma
Confetti plots illustrating the spatial distribution of cell subsets are shown for each ROI included in this study. Each image shows segmented cells, color-coded to distinguish immune and non-immune cell subsets.

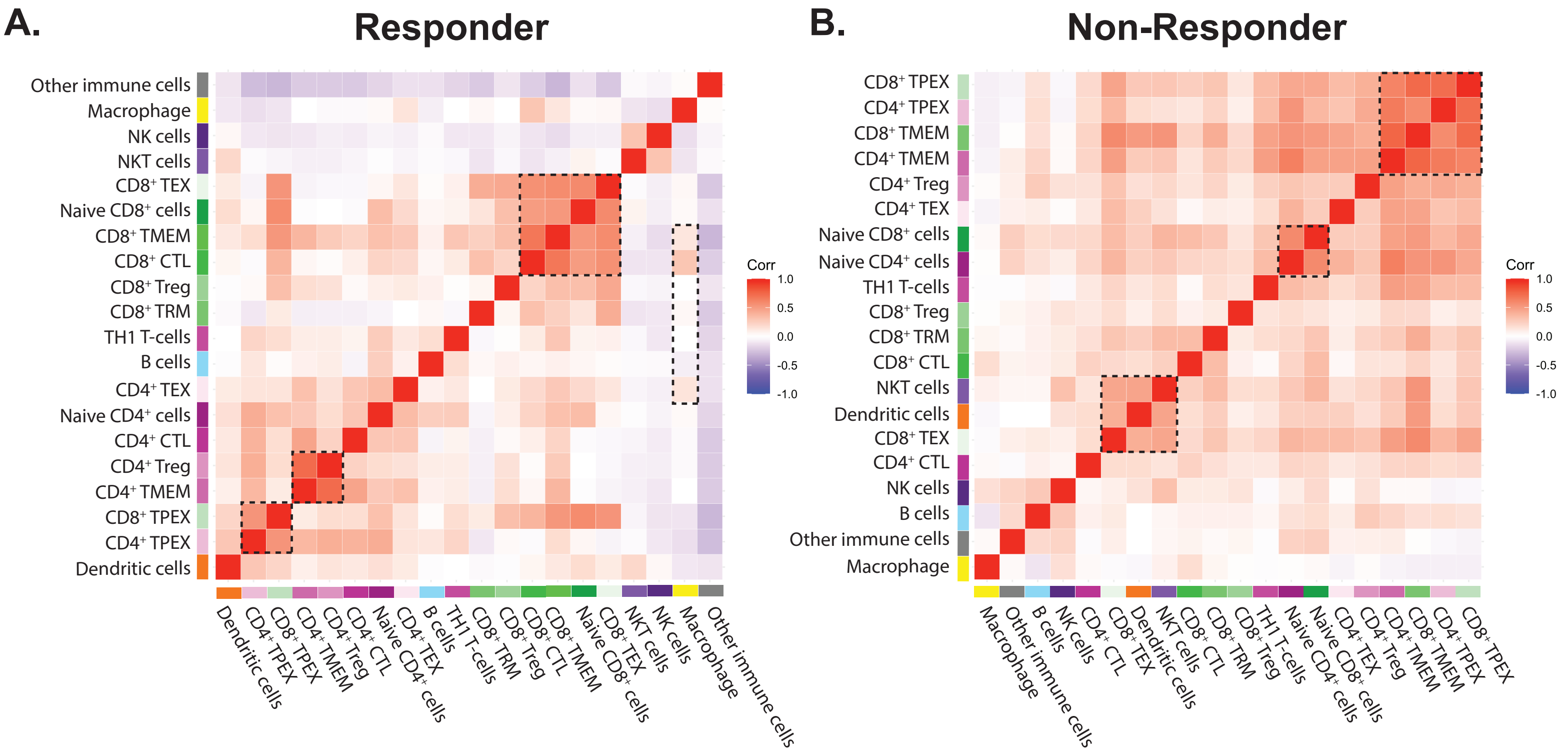

Supplementary Figure 13. Neighborhood analysis of T cells in metastatic melanoma.
A. Correlation heatmap showing the correlation of spatial localization of T cell subsets within 50 mm raster-based defined neighborhoods in responders. Colors on the borders of the plot indicate the annotated cell lineages. Pearson correlation tests indicate avoidance (blue) or co-localization (red) of cellular localization. The boxes indicate specific correlation patterns which are explained in the text. **B.** Correlation heatmap showing the correlation of spatial localization of T cell subsets within 50 mm raster-based defined neighborhoods in non-responders. Colors on the borders of the plot indicate the annotated cell lineages. Pearson correlation tests indicate avoidance (blue) or co-localization (red) of cellular localization. The boxes indicate specific correlation patterns which are explained in the text.
